# AtNHX5 and AtNHX6 Are Required for the Subcellular Localization of the SNARE Complex That Mediates the Trafficking of Seed Storage Proteins in *Arabidopsis*

**DOI:** 10.1371/journal.pone.0151658

**Published:** 2016-03-17

**Authors:** Xuexia Wu, Kazuo Ebine, Takashi Ueda, Quan-Sheng Qiu

**Affiliations:** 1 MOE Key Laboratory of Cell Activities and Stress Adaptations, School of Life Sciences, Lanzhou University, Lanzhou, Gansu, 73000, China; 2 Department of Biological Sciences, Graduate School of Sciences, The University of Tokyo, 7-3-1 Hongo, Bunkyo-ku, Tokyo, 113-0033, Japan; 3 Japan Science and Technology Agency (JST), PRESTO, 4-1-8 Honcho Kawaguchi, Saitama, 332-0012, Japan; Beijing Forestry University, CHINA

## Abstract

The SNARE complex composed of VAMP727, SYP22, VTI11 and SYP51 is critical for protein trafficking and PSV biogenesis in *Arabidopsis*. This SNARE complex directs the fusion between the prevacuolar compartment (PVC) and the vacuole, and thus mediates protein trafficking to the vacuole. In this study, we examined the role of AtNHX5 and AtNHX6 in regulating this SNARE complex and its function in protein trafficking. We found that AtNHX5 and AtNHX6 were required for seed production, protein trafficking and PSV biogenesis. We further found that the *nhx5 nhx6 syp22* triple mutant showed severe defects in seedling growth and seed development. The triple mutant had short siliques and reduced seed sets, but larger seeds. In addition, the triple mutant had numerous smaller protein storage vacuoles (PSVs) and accumulated precursors of the seed storage proteins in seeds. The PVC localization of SYP22 and VAMP727 was repressed in *nhx5 nhx6*, while a significant amount of SYP22 and VAMP727 was trapped in the Golgi or TGN in *nhx5 nhx6*. AtNHX5 and AtNHX6 were co-localized with SYP22 and VAMP727. Three conserved acidic residues, D164, E188, and D193 in AtNHX5 and D165, E189, and D194 in AtNHX6, were essential for the transport of the storage proteins, indicating the importance of exchange activity in protein transport. AtNHX5 or AtNHX6 did not interact physically with the SNARE complex. Taken together, AtNHX5 and AtNHX6 are required for the PVC localization of the SNARE complex and hence its function in protein transport. AtNHX5 and AtNHX6 may regulate the subcellular localization of the SNARE complex by their transport activity.

## Introduction

Seeds of crop plants are the principle source of proteins for humans and livestock [1–3]. Seed storage proteins are primarily accumulated in the protein storage vacuoles (PSVs) [4–7]. Thus, understanding the mechanism underlying the trafficking of the storage proteins to the PSVs is critical for improvements in the protein yield as well as protein quality of the crop seeds [2, 3].

Seed storage proteins are synthesized as precursors in the endoplasmic reticulum (ER). They are then transported into the PSVs where the precursors are converted to mature forms [8–10]. Two distinct pathways have been identified for the trafficking of storage proteins to the PSVs: the Golgi-dependent and -independent pathways [11–14]. In the Golgi-dependent pathway, proteins are transported through the Golgi and then dense vesicles before being delivered into the PSVs [15–19]. In the Golgi-independent pathway, however, proteins are transported from the ER to the PSVs through the precursor-accumulating (PAC) vesicles [11, 13].

The molecular machinery involved in protein trafficking to the PSVs is beginning to be explored. VSR1, VSR3 and VSR4 have been shown to function as the vacuolar sorting receptors (VSR) of the PSVs in *Arabidopsis* [20–22]. RMR1, the receptor homology region transmembrane domain ring H2 motif protein, is another type of sorting receptors for the trafficking to the PSVs in *Arabidopsis* [19, 23]. PV72 was reported to be the pumpkin VSR on the membranes of the PAC vesicles [24]. AtVPS29, a member of the retromer complex that recycles VPS10 from the prevacuolar compartment (PVC) to the Golgi, is involved in recycling VSRs for the sorting of storage proteins [7, 25]. In addition, the SNARE complex composed of VAMP727, SYP22, VTI11, and SYP51, which mediates the fusion between the PVC and the vacuole, is crucial for protein transport into the PSVs in *Arabidopsis* [26].

Cellular pH is a key regulatory factor of protein trafficking in both the secretory and endocytic pathways [27–30]. Studies have shown that the organelles become more acidic along the process of maturation in the exocytic or endocytic pathways in both the plant and animal cells [27, 28, 31]. In yeast and animals, the acidity of the organelles is generated by the vacuolar-type H^+^-ATPases (V-ATPases) [27, 32]. Moreover, Na^+^/H^+^ antiporters (NHXs) in animals conduct proton leak to counter organelle acidification in order to maintain an optimal pH [33, 34]. In plants, however, the acidic pH of the organelles is maintained by the proton pumps V-ATPases and pyrophosphatase [30, 31]. Similar to their animal counterparts, the Golgi/TGN-localized plant NHX antipoters, AtNHX5 and AtNHX6, may act as a H^+^-leak system to counter the luminal acidification [31].

Studies have shown that protein trafficking to the PSVs is controlled by pH and requires V-ATPases and NHX antiporters: (1) The binding of the VSR to its ligand is pH-dependent; the binding occurs from pH 4.0 to 7.0, with an optimal binding at pH 6.0 [35]; (2) V-ATPase is required for the sorting of soluble vacuolar protein precursors in tobacco cells [36]; (3) The antibiotic Na^+^/H^+^ antiporter monensin, which causes the acidification of the TGN, affects the vacuolar transport of the seed storage proteins [37, 38]; and (4) AtNHX5 and AtNHX6 are required for the transport of the seed storage proteins into the vacuole as well as processing of the seed storage proteins in *Arabidopsis* [39,40].

Plant NHX antiporters are membrane proteins that transport protons (H^+^) across a membrane in exchange for Na^+^ or K^+^. Studies show that plant NHX antiporters are critical for cellular ion homeostasis and pH regulation, and play significant roles in diverse cellular processes, including pH homeostasis, Na^+^ and K^+^ movement, vesicle trafficking and fusion, growth and development, and salt tolerance [41–45]. The *Arabidopsis* NHX gene family contains 8 members that are divided into three distinct classes: vacuolar (AtNHX1-AtNHX4), endosomal (AtNHX5 and AtNHX6), and plasma membrane (AtNHX7/SOS1and AtNHX8) [41, 43, 46]. AtNHX5 and AtNHX6 are localized in the Golgi, TGN, and PVC, and share high sequence similarity (78.7%) [39, 41, 47]. AtNHX5 and AtNHX6 play an important role in plant growth and development [48, 49]. *nhx5 nhx6* double mutant showed profound defects in growth and development. *nhx5 nhx6* had smaller rosettes and shorter seedlings, and was flowering and bolting late [48, 49]. These results demonstrate that AtNHX5 and AtNHX6 are essential for the growth and development in *Arabidopsis*.

How the trafficking of seed storage proteins is controlled by AtNHX5 and AtNHX6, however, remains unclear. Reguera et al. (2015) found that the binding of VSR to its cargoes was reduced in *nhx5 nhx6* [39]. This discovery suggested that AtNHX5 and AtNHX6 might function in controlling the interaction between VSR and its cargoes [39]. In addition, Ashnest et al. (2015) showed that AtNHX6 interacted with SNX1, a component of the Retromer. Retromer is the cellular sorting machinery that recycles VSRs back to the ER from the TGN [40]. Therefore, these findings from Ashnest et al. (2015) suggest that AtNHX5 and AtNHX6 might regulate the recycling of VSRs. Considering that AtNHX5 and AtNHX6 are localized in Golgi, TGN, and PVC, key players of the protein trafficking pathway, their function may not be limited to regulating VSR binding activity [4, 39, 50]. They may have diversified function. As mentioned above, the four SNAREs, VAMP727, SYP22, VTI11, and SYP51, play a crucial role in vacuolar transport, seed maturation, and vacuole biogenesis [26]. They form a complex that mediates the fusion between the PVC and the vacuole, and through which proteins are delivered into the vacuole [26]. Notably, these four SNAREs are localized to the PVC, the same organelle where AtNHX5 and AtNHX6 reside. Then, it is interesting to understand whether this SNARE complex is regulated by AtNHX5 and AtNHX6.

In this study, we aim to examine the role of AtNHX5 and AtNHX6 in regulating the SNARE complex of VAMP727, SYP22, VTI11, and SYP51. We started with genetic analysis for the double mutant *nhx5 nhx6* and the triple mutant *nhx5 nhx6 syp22*. We found that AtNHX5 and AtNHX6 were required for seed production, protein trafficking and PSV biogenesis. We further found that the triple mutant showed severe defects in seedling growth and seed development. The PSV was smaller but its number was increased in the triple mutant. The precursors of the seed storage proteins were accumulated in the triple mutant. We further found that the PVC localization of the SNARE proteins SYP22 and VAMP727 was repressed in *nhx5 nhx6*. We identified three conserved acidic residues that were essential for the transport of the storage proteins. But AtNHX5 and AtNHX6 did not interact physically with these SNARE molecules. These results suggest that AtNHX5 and AtNHX6 regulate the subcellular localization of the SNARE complex and thus its function in protein transport.

## Results

### Seed production is altered in *nhx5 nhx6*

In order to investigate the role of AtNHX5 and AtNHX6 in controlling the trafficking of the seed storage proteins, we examined silique growth and seed production of the *nhx5 nhx6* double mutant. The *nhx5 nhx6* double mutants were generated in our previous study [49]. Briefly, we obtained one T-DNA line for the *AtNHX5* gene (*nhx5-1*) and two separate T-DNA lines for the *AtNHX6* gene (*nhx6-1* and *nhx6-2*) [49]. The double knockout lines were produced by crossing *nhx5-1* with *nhx6-1* or *nhx6-2*, respectively, to obtain two independent double knockout lines, *nhx5-1 nhx6-1* and *nhx5-1 nhx6-2*. The absence of the *AtNHX5* and *AtNHX6* transcripts in these double knockout lines was confirmed by RT-PCR [49]. These two double knockout lines had identical growth phenotypes [49]. The *nhx5-1 nhx6-1* double mutant line was used in the following experiments.

**Fig 1:**
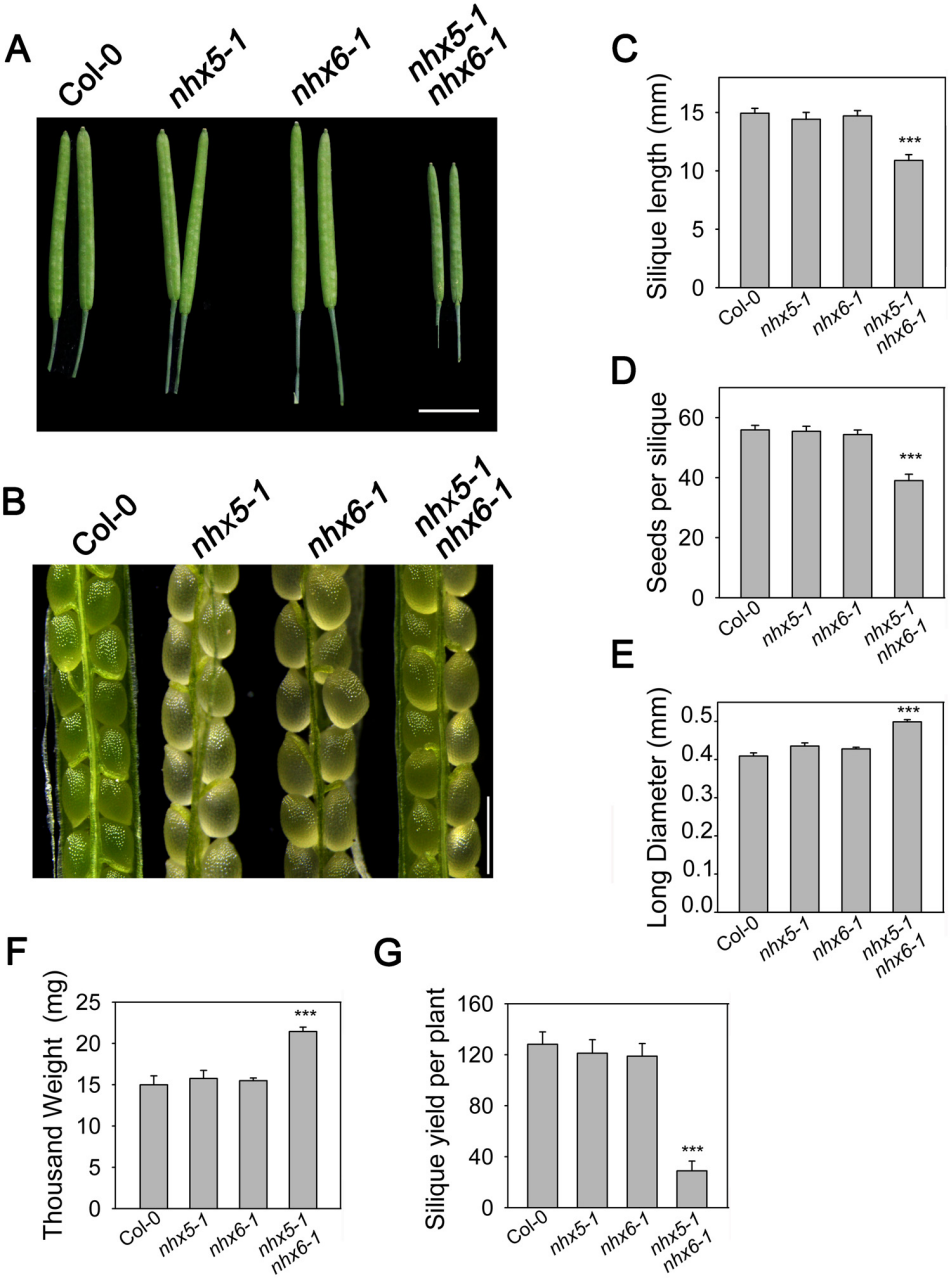
Seed production was altered in *nhx5 nhx6*. (A) Silique phenotype. Photos were taken for siliques on the main stem at 22 DAF. Bar = 0.5 cm. (B) Embryo morphology at 14 DAF. Bars = 1mm. (C) Silique length. Length of siliques on the main stem was measured (average length of 6 siliques) at 22 DAF. Error bars represent SD; n = 15. (D) Seed yield per silique. The total seeds per silique were counted (average number of 6 siliques). Error bars represent SD, n = 15. (E) Long diameter of dry seeds. Error bars represent SD, n = 250. (F) Thousand grain weight of seeds. Error bars represent SD, n = 8. (G) Silique yield per plant. The total siliques of each plant were counted. Error bars represent SD, n = 15. ***P≤0.001, by *t* test.

Interestingly, besides the profound defects in growth and development, *nhx5 nhx6* double mutants had smaller siliques (Fig 1A). The siliques of *nhx5 nhx6* were 26% shorter than that of the wild-type plants (Fig 1C). Additionally, *nhx5 nhx6* produced less siliques (Fig 1G) and contained less seeds (Fig 1D). The seed yield per silique of *nhx5 nhx6* was reduced 29% compared to the wild-type plants (Fig 1D). Similar to what Reguera et al. (2015) reported, we found that *nhx5 nhx6* produced larger seeds (Fig 1B and 1E). The seeds of *nhx5 nhx6* were 21% longer than that of the wild-type plants (Fig 1E). The thousand grain weight of *nhx5 nhx6* was increased by 43% compared with the wide-type plants (Fig 1F). But the morphology of the mature seeds still looked the same as the wild type plants (Fig 1B). These results suggest that AtNHX5 and AtNHX6 play an important role in seed growth and development in *Arabidopsis*. However, there was no significant difference between the single mutant *nhx5* or *nhx6* and the wild type plants in silique growth and seed production (Fig 1), suggesting that *AtNHX5* and *AtNHX6* are functionally redundant.

### The artificial seed protein GFP-CT24 is missorted in *nhx5 nhx6*

We then examined if AtNHX5 and AtNHX6 controlled the transport of seed storage proteins to the PSVs. We used the artificial protein GFP-CT24 as a marker to track protein trafficking toward the PSVs in the embryo of the mature seeds. The artificial protein GFP-CT24 is produced by expressing the construct SP-GFP-CT24 in *Arabidopsis*. SP-GFP-CT24 consists of a signal peptide, GFP, and the C-terminal 24 amino acids (CT24) of the α’ subunit of β-conglycinin [10, 51]. β-conglycinin, a 7S globulin, is one of the major storage proteins in soybean seeds. CT24 contains the vacuolar sorting determinant of α’ subunit. Studies have shown that the artificial protein GFP-CT24 can be expressed and transported into the PSVs in the seeds of *Arabidopsis* and soybean [10, 51].

**Fig 2:**
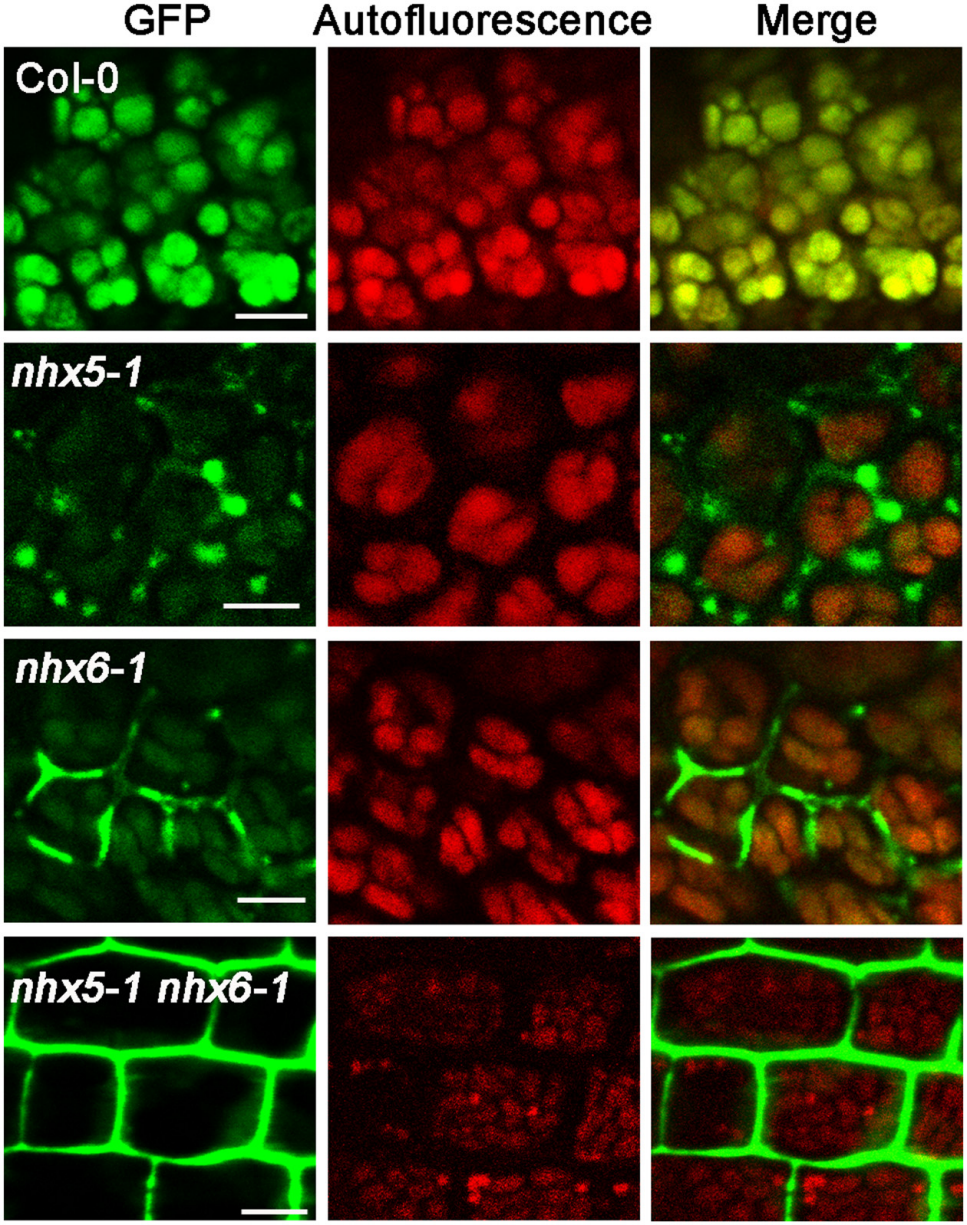
The artificial protein GFP-CT24 is missorted out of the cells of the mature seeds in *nhx5 nhx6*. The artificial seed protein GFP-CT24 was used as a marker to track protein trafficking to the PSVs in the embryos of the mature seeds. GFP-CT24 was expressed in *Arabidopsis* seedlings and GFP-CT24 fluorescent signal was visualized with a confocal laser scanning microscope. Bars = 10 μm.

To track protein trafficking with GFP-CT24, we generated the transgenic seedlings expressing the artificial seed protein GFP-CT24. The transformants were produced in the backgrounds of wild-type, single mutant (*nhx5* or *nhx6*) and double mutant (*nhx5 nhx6*) plants (Fig 2). We then compared the expression and transport of GFP-CT24 in the seeds of the mutant plants with that of the wild-type plants. GFP-CT24 fluorescent signals were visualized with a confocal laser scanning microscope. In the seeds of wild-type plants, GFP fluorescence predominantly appeared in the PSVs (Fig 2), indicating that the artificial seed protein GFP-CT24 was expressed and transported effectively into the PSVs in the wild-type plants. However, in either *nhx5* or *nhx6* single mutants, a strong GFP fluorescence signal was observed in the extracellular spaces, while a relatively faint signal of GFP fluorescence appeared in the PSVs, indicating that protein transport is impaired in *nhx5* or *nhx6* single mutants (Fig 2). Interestingly, GFP fluorescence predominantly appeared in the extracellular spaces in *nhx5 nhx6*, indicating that the artificial seed protein GFP-CT24 is missorted out of the cells in *nhx5 nhx6* (Fig 2). These results suggest that AtNHX5 and AtNHX6 may control the transport of the seed storage proteins to the PSVs in *Arabidopsis*.

We next examined the profile of seed storage proteins using SDS-PAGE and immunoblot. We found that *nhx5 nhx6* accumulated a significant amount of the precursor proteins of both 12S globulin and 2S albumin (S1 Fig). This result was verified by examining the distribution of 12S globulin in the embryo of the mature seeds using a transmission electron microscopy. The immune EM showed that a striking amount of the unprocessed precursors of 12S globulin were detected in the intercellular space of *nhx5 nhx6* (S2 Fig). In addition, we found that PSV’s size is reduced but its number is increased in *nhx5 nhx6*, suggesting that AtNHX5 and AtNHX6 may involve in the biogenesis of the PSVs in *Arabidopsis* (S3 Fig).

All these results are similar to the findings of Reguera et al. (2015) [39]. These results confirmed the notion that AtNHX5 and AtNHX6 are required for the transport of the seed storage proteins into the PSVs as well as the biogenesis of the PSVs in *Arabidopsis* [39].

### Three conserved acidic residues in AtNHX5 and AtNHX6 are critical for the transport of seed storage proteins into the PSVs in *Arabidopsis*

Our previous study has shown that AtNHX5 and AtNHX6 contain the conserved acidic amino acid residues in transmembrane domains that align with the yeast Na^+^/H^+^ antiporter ScNhx1p [49]. These conserved acidic residues of AtNHX5 (D164, E188, D193 and E320) and AtNHX6 (D165, E189, D194 and E320) line up with the corresponding residues in ScNhx1p (D201, E225, D230 and E355) [49, 52]. We have shown that three of these conserved acidic residues of AtNHX5 (D164, E188, and D193) and AtNHX6 (D165, E189, and D194) are essential for the ion transport activity of AtNHX5 and AtNHX6, and thus play an important role in growth and development in *Arabidopsis*. We are then asking whether the transport activity of AtNHX5 and AtNHX6 is required for the transport of seed storage proteins.

**Fig 3:**
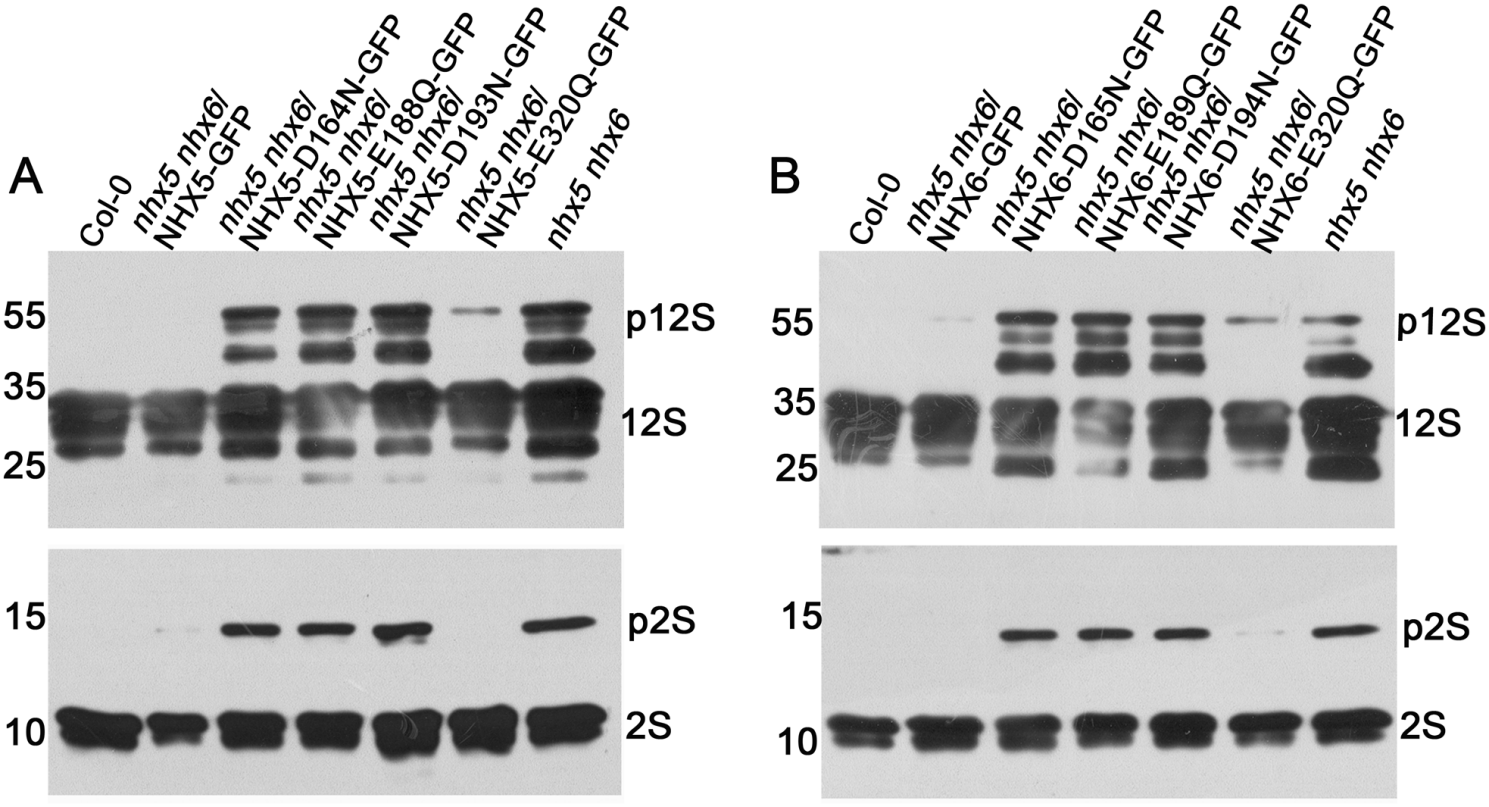
Three conserved acidic residues in AtNHX5 and AtNHX6 are critical for the transport of the storage proteins into the PSVs in Arabidopsis. (A) and (B) Immunoblot analysis for the point mutations in AtNHX5 (A) and AtNHX6 (B). The point mutated genes of the four conserved acidic residues of AtNHX5 and AtNHX6 were introduced into the *nhx5 nhx6* background, respectively. Total proteins were extracted from the mature seeds. 10 μg proteins were loaded in each lane. Proteins were probed with anti-12S globulin and anti-2S albumin antibodies, respectively.

To this end, we expressed *AtNHX5* and *AtNHX6* genes mutated in these four conserved residues in *nhx5 nhx6*. The mutations were made by replacing the acidic residues with the uncharged polar residues. In AtNHX5, D164 was mutated to N, E188 to Q, D193 to N and E320 to Q; in AtNHX6, D165 to N, E189 to Q, D194 to N and E320 to Q. Protein profiles of the mature seeds were analyzed by SDS-PAGE and immunoblot (Fig 3). No precursors were detected for the control plants expressing the wild type *AtNHX5* or *AtNHX6* gene (Fig 3A and 3B), indicating that the transformed *AtNHX5* or *AtNHX6* gene functions efficiently in the transport of the seed storage proteins. However, the precursor proteins of both 12S globulin and 2S albumin were detected for three of the point mutations in both *AtNHX5* and *AtNHX6* genes (D164N, E188Q and D193N of AtNHX5, and D165N, E189Q and D194N of AtNHX6) (Fig 3A and 3B), indicating that these mutant genes were malfunctioning in protein transport. These results suggest that the transport activity of AtNHX5 and AtNHX6 is required for the transport of seed storage proteins.

Our previous study has shown that E320 in both *AtNHX5* and *AtNHX6* genes may not be involved in exchange activity and cellular functions, since E320Q mutant remained its activity in conferring yeast growth under both high K^+^ and hygromicin B, and E320Q mutant fully recovered the growth of the *nhx5 nhx6* seedlings [49]. As expected, E320Q mutant accumulated no or little precursors for both *AtNHX5* and *AtNHX6* genes (Fig 3A and 3B), indicating that protein transport was not or partially impaired in E320Q.

### Seedling growth and seed development are altered in *nhx5 nhx6 syp22*

Since AtNHX5, AtNHX6 and the SNAREs VAMP727, SYP22, VTI11 and SYP51 are all localized to the same organelle PVC, we reason that AtNHX5 and AtNHX6 may control the trafficking of seed storage proteins by regulating the function of this SNARE complex.

**Fig 4:**
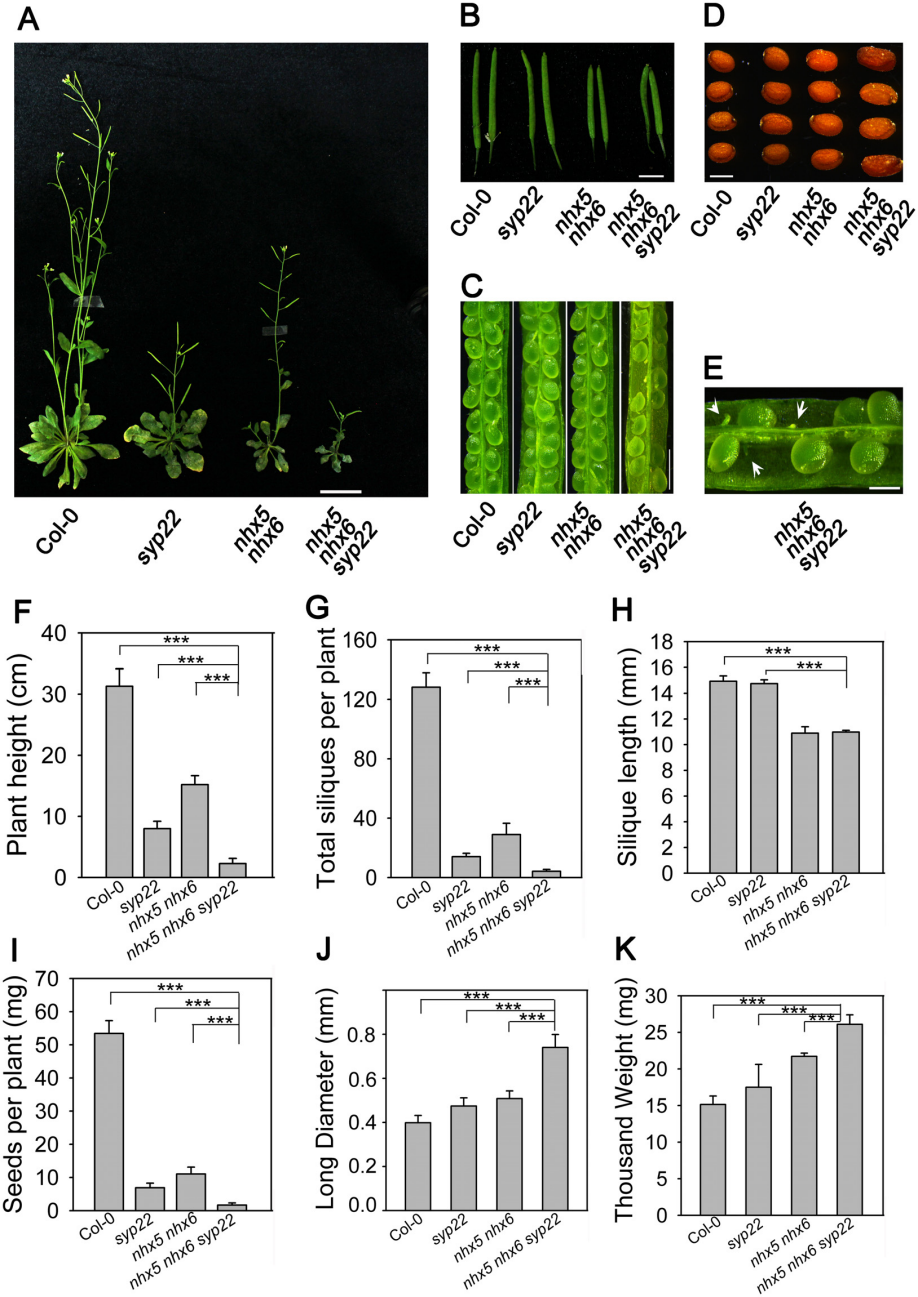
Seedling growth and seed development are altered in *nhx5 nhx6 syp22*. (A) Phenotype of the triple mutant grown on soil for 60 days. (B) Silique phenotype. Photos were taken at 22 DAF. Bar = 0.5 cm. (C) The embryo morphology at 14 DAF. Bars = 1mm. (D) The mature seeds. Photos were taken when the seeds were dehydrated for 30 days after harvest. Bars = 0.5mm. (E) Embryo morphology of the triple mutant. Bars = 0.5mm. (F) Plant height. Error bars represent SD; n = 30. (G) Silique yield per plant. Error bars represent SD; n = 30. (H) Silique length. Silique length was measured on the main stem at 22 DAF. Error bars represent SD; n = 15. (I) Seed yield per silique. The total seeds per silique were counted on the main stem. Error bars represent SD, n = 15. (J) Long diameter of the dry seeds. Error bars represent SD. n = 300. (K) Thousand weight of the seeds. Error bars represent SD, n = 8. ***P≤0.001, by *t* test.

To test this hypothesis, we generated the *nhx5 nhx6 syp22* triple mutant (Fig 4). We obtained two separate T-DNA lines for the *SYP22* gene (*syp22-1* and *syp22-3*) (S4A Fig). The *nhx5-1 nhx6-1* double mutant line was generated in our previous study [49]. The double mutant line *nhx5-2 nhx6-2* was generated by crossing *nhx5-2* and *nhx6-2*. The triple mutant lines were generated by crossing *nhx5-1 nhx6-1* with *syp22-3*, and *nhx5-2 nhx6-2* with *syp22-1*, respectively, to obtain two independent triple knockout lines, *nhx5-1 nhx6-1 syp22-3* and *nhx5-2 nhx6-2 syp22-1*. The absence of *AtNHX5* and *AtNHX6* transcripts in the double knockout lines was confirmed by RT-PCR (S4C Fig; 49). The absence of *AtNHX5*, *AtNHX6* and *SYP22* transcripts in triple knockout lines was confirmed by RT-PCR (S4D Fig). These two triple knockout lines had identical growth phenotypes (S4B Fig). The *nhx5-1 nhx6-1 syp22-3* triple mutant line was used in the following experiments.

Studies have found that the *nhx5 nhx6* double mutant showed profound defects in growth and development (Fig 4A) [48, 49]. *nhx5 nhx6* had smaller rosettes and shorter seedlings, was flowering and bolting late, and produced less seeds (Fig 4A) [48, 49]. In addition, the *syp22* mutant is impaired in growth and development (Fig 4A) [53, 54]. *syp22* showed multiple abnormal phenotypes, including semi-dwarfism, serrated wavy leaves, late flowering, and resistance to salt stress (Fig 4A) [53, 54]. Interestingly, the *nhx5 nhx6 syp22* triple mutant showed much more severe defects in growth and development (Fig 4A). The triple mutant was smaller than *nhx5 nhx6* or *syp22* (Fig 4A). The height of the triple mutant seedlings was 13%, 40% and 26% of the wild type, *syp22*, and *nhx5 nhx6* seedlings, respectively, when grown in soil for 60 days (Fig 4A and 4F). The triple mutant had short siliques and reduced seed sets, and contained approximately 24% aborted seeds (Fig 4B, 4C and 4E). The triple mutant produced less siliques and seeds (Fig 4G and 4I). However, the triple mutant produced much larger seeds (Fig 4D). The seed of the triple mutant was 85%, 56%, and 45% larger in length than the wild type, *syp22*, and *nhx5 nhx6*, respectively (Fig 4D and 4J). The thousand grain weight of the triple mutant was increased by 73%, 53% and 23%, respectively, comparing with the wild type, *syp22* and *nhx5 nhx6* (Fig 4K). The severe defects in seedling growth and seed production of the triple mutant suggest that AtNHX5, AtNHX6 and SYP22 have an overlapped function in growth and development, particularly in silique growth and seed development.

### PSV’s size is reduced but its number is increased in *nhx5 nhx6 syp22*

**Fig 5:**
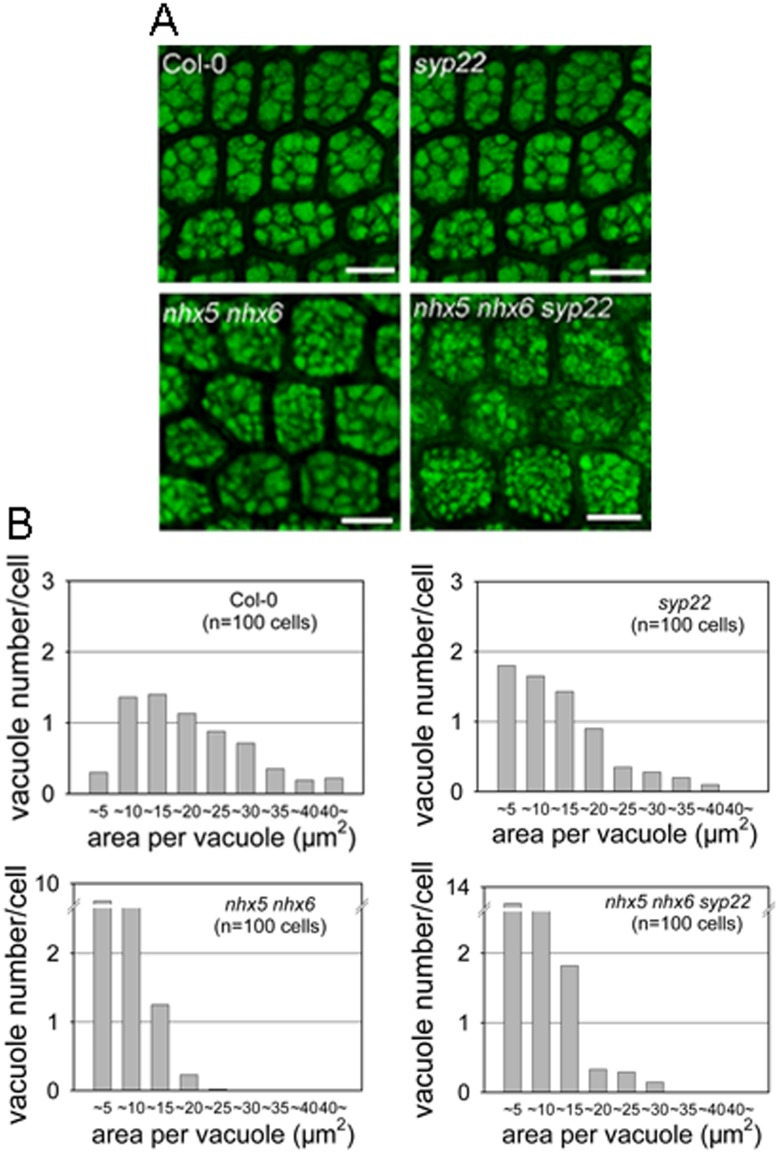
AtNHX5, AtNHX6 and AtSYP22 play an important role in the biogenesis of the PSVs in Aabidopsis. (A) Morphology of the PSVs. Autofluorescence of the PSVs was visualized with a confocal laser scanning microscope. Bars = 10 μm. (B) Histograms representing a distribution of size and number of vacuoles within a single cell. The X axis indicates the areas of the vacuole. Area of each vacuole was measured from the embryo cells of Col-0 and mutants. 100 cells were measured for PSV analysis.

In order to understand whether AtNHX5, AtNHX6 and SYP22 control the biogenesis of the PSVs in *Arabidopsis*, we analyzed the morphology of the PSVs (Fig 5). The autofluorescence of the PSVs was visualized under a confocal microscope (Fig 5A). For the wild-type plants, the average section area per vacuole is 17.55 ± 6.38 μm^2^ (mean ± SD; n = 654 PSVs in 100 cells) (Fig 5A and 5B). The PSVs in *syp22* was slightly reduced: 10.42 ± 8.5 μm^2^ (mean ± SD; n = 665 PSVs in 100 cells) (Fig 5A and 5B). The PSVs in *nhx5 nhx6* were fragmented and showed a significant reduction in average vacuole size: 5.5 ± 3.12 μm^2^ (mean ± SD; n = 1647 PSVs in 100 cells) (Fig 5A and 5B). Interestingly, the triple mutant had a much smaller PSV with an average size of 4.5±2.12 μm^2^ (mean ± SD; n = 2400 PSVs in 100 cells) (Fig 5A and 5B).

In addition, the number of the PSVs in the triple mutant was increased significantly. While the average number of the PSVs is 6.5 in wild-type plants, *syp22* and *nhx5 nhx6* had an average of 6.6 and 16 PSVs, respectively (Fig 5A and 5B). However, the triple mutant had a significantly increased number of PSVs: 24 PSVs per embryo cell (Fig 5A and 5B). These results suggest that AtNHX5, AtNHX6 and SYP22 may involve in the biogenesis of the PSVs in *Arabidopsis*.

### Precursors of seed storage proteins are accumulated in *nhx5 nhx6 syp22*

12S globulin and 2S albumin are the two major storage proteins of the PSVs in *Arabidopsis* [10, 20]. They are synthesized as precursors in endoplasmic reticulum and sorted to the PSVs to convert to the mature proteins [20]. It is interesting to ask whether AtNHX5, AtNHX6 and SYP22 control the transport of 12S globulin and 2S albumin into the PSVs in *Arabidopsis*.

**Fig 6:**
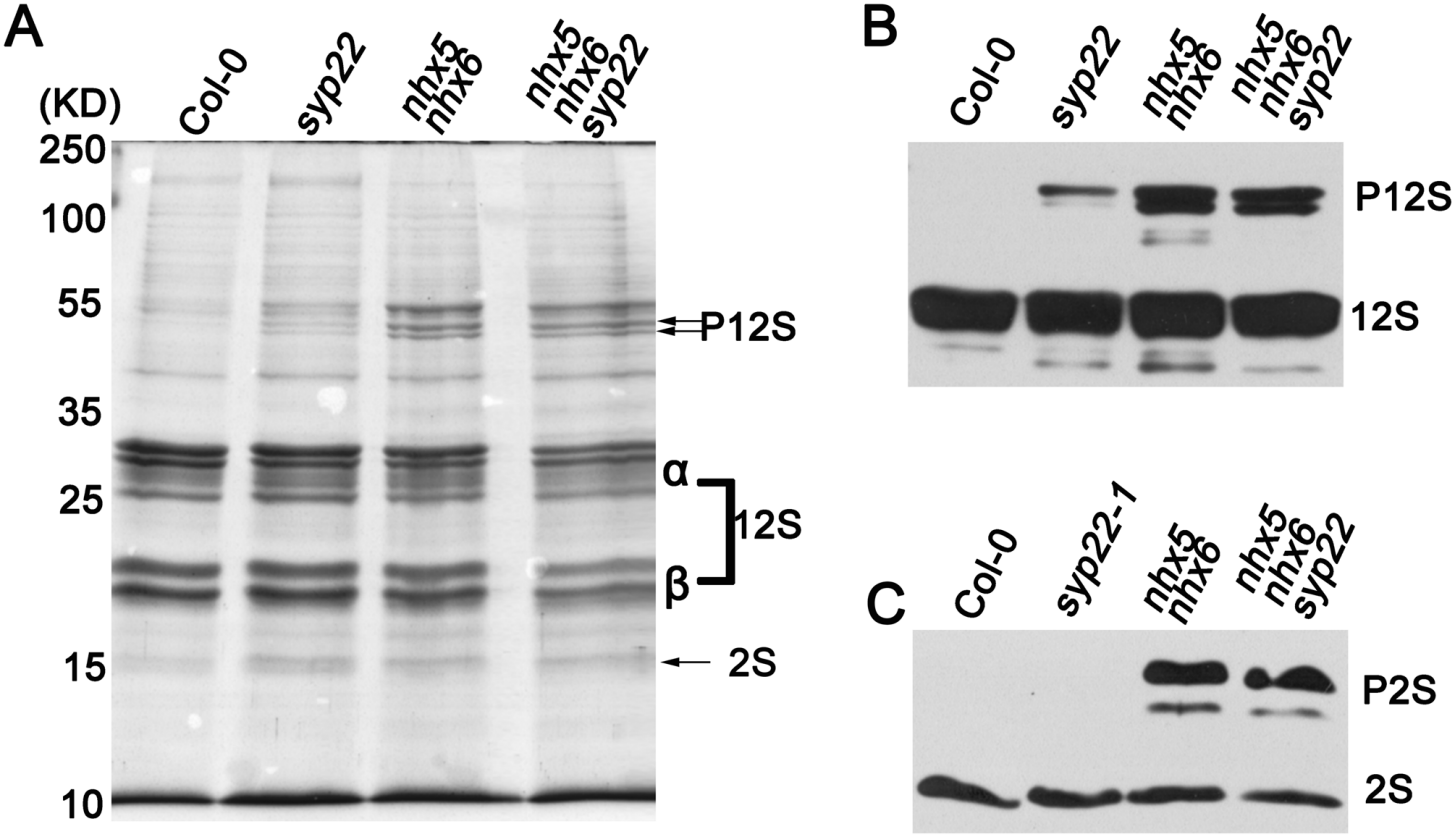
Precursors of the storage proteins are accumulated in the seeds of *nhx5 nhx6 syp22*. (A) SDS-PAGE stained with the Coomassie blue. Total proteins were extracted from the mature seeds. 10 μg proteins were loaded in each lane. (B) and (C) Immunoblot analysis of 12S globulin (B) and 2S albumin (C).

To this end, we examined the profile of seed storage proteins using SDS-PAGE and immunoblot. Total proteins were extracted from the mature seeds of wild-type, *syp22*, *nhx5 nhx6*, and *nhx5 nhx6 syp22* seedlings, respectively. The proteins were separated on SDS-PAGE, stained with coomassie blue, and blotted with anti-12S or anti-2S antibodies (Fig 6). A high amount of the mature forms of 12S globulin and 2S albumin were detected in the seeds of wild-type plants; but no precursor proteins were detected (Fig 6A to 6C), indicating that 12S globulin and 2S albumin were transported into the PSVs and converted to their mature forms in wild-type plants. A faint band of the precursor proteins of 12S globulin was detected in the seeds of *syp22*; but the precursors of 2S albumin was not detected in *syp22* (Fig 6A to 6C). Interestingly, *nhx5 nhx6* and *nhx5 nhx6 syp22* accumulated a significant amount of the precursor proteins of both 12S globulin and 2S albumin, while the mature forms of 12S globulin and 2S albumin were slightly reduced (Fig 6A to 6C). These results suggest that the transport of 12S globulin and 2S albumin was partially impaired, and 12S globulin and 2S albumin were missorted out of the cells in *nhx5 nhx6 syp22*.

### Subcellular localization of SYP22 and VAMP727 is altered in *nhx5 nhx6*

In plant cells, proteins are transported to the vacuole through a vesicle-mediated trafficking pathway that includes the ER, Golgi, TGN, and MVB/PVC [50]. Thus, the Golgi, TGN and MVB/PVC are major protein sorting stations in vacuolar transport [55, 56]. Since AtNHX5 and AtNHX6 are localized to the Golgi, TGN and PVC, these two antiporters may involve in protein trafficking towards the vacuole [39]. Studies have shown that the SNARE complex composed of VAMP727, SYP22, VTI11 and SYP51 was localized to PVC to mediate the fusion between the PVC and vacuole [26]. Then, it is interesting to ask whether AtNHX5 and AtNHX6 are required for the trafficking and localization of the SNAREs VAMP727, SYP22, VTI11 and SYP51.

**Fig 7:**
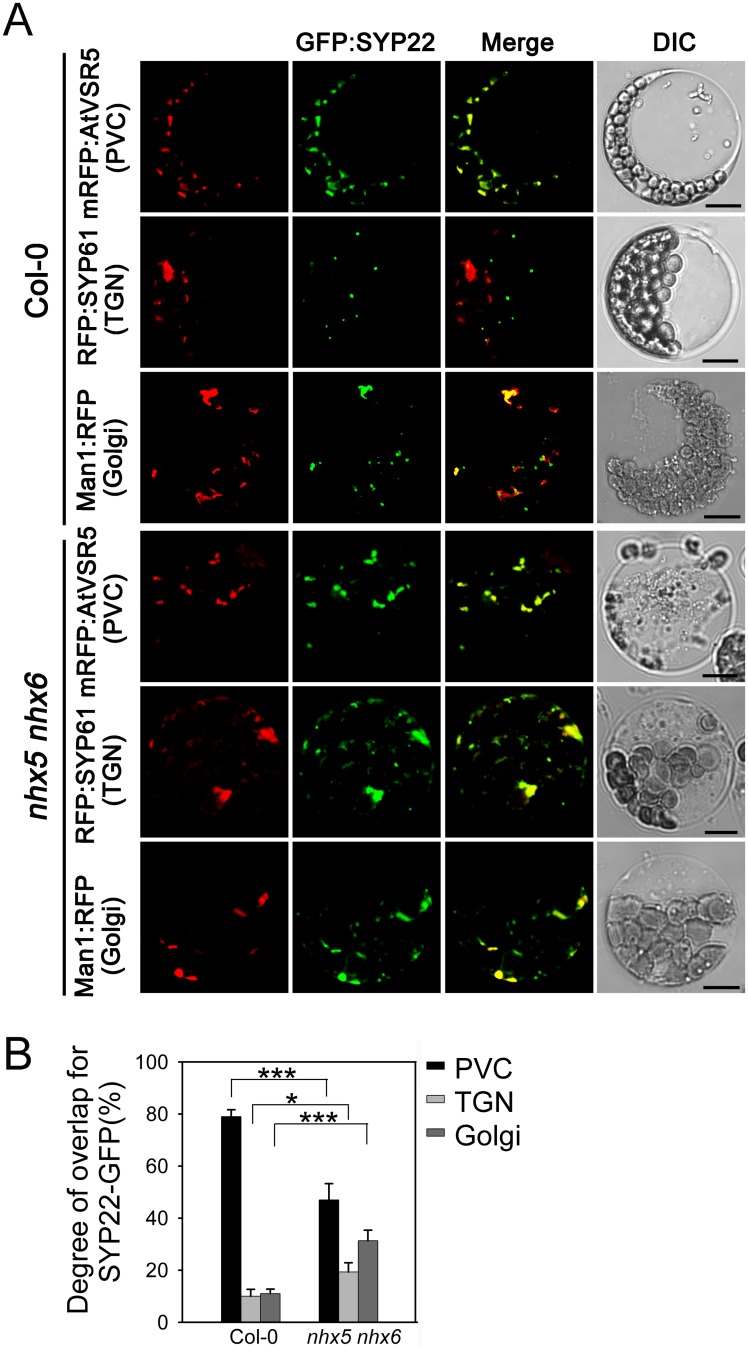
Subcellular localization of AtSYP22 is altered in *nhx5 nhx6*. (A) Subcellular localization of GFP:SYP22. The *SYP22*:*GFP* plasmid was co-transformed with the PVC marker mRFP:AtVSR5, TGN marker RFP:SYP61 or Golgi marker Man1:RFP into the leaf protoplasts of Arabidopsis, respectively. Fluorescence was visualized by a confocal laser scanning microscope. Bar = 10μm. (B) Quantification of the subcellular localization pattern of AtSYP22. The overlapping percentage of GFP:SYP22 signal with mRFP:AtVSR5, RFP:SYP61 or Man1:RFP, respectively, was determined from more than 100 protoplasts obtained from three independent experiments. Error bars represent SD, n = 3. ***P≤0.001, *P≤0.05, by *t* test.

**Fig 8:**
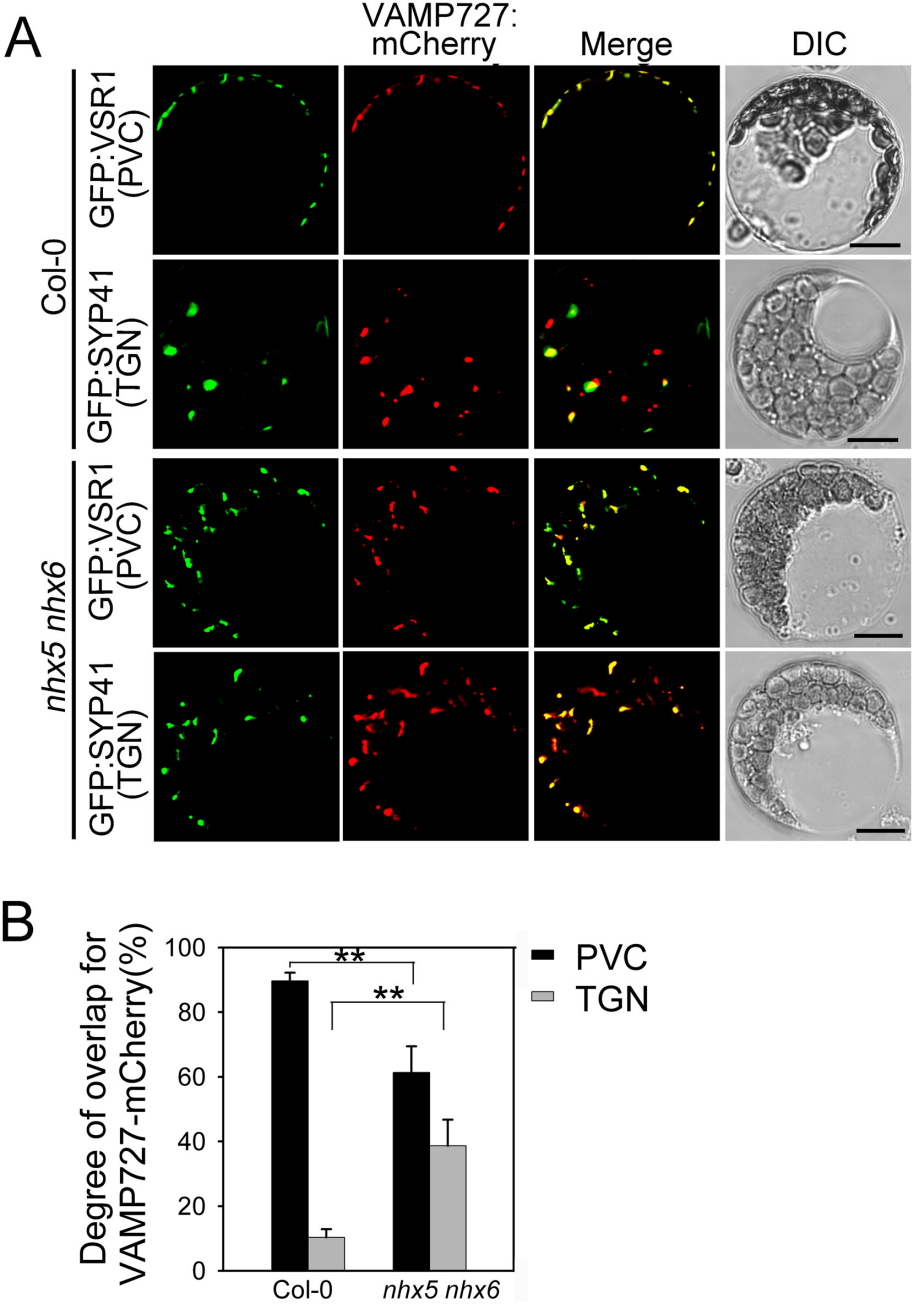
Subcellular localization of AtVAMP727 is altered in *nhx5 nhx6*. (A) Subcellular localization of VAMP727:mCherry. The *VAMP727*:*mCherry* plasmid was co-transformed with the PVC marker GFP:AtVSR1 and TGN marker GFP:SYP41, respectively, into the leaf protoplasts of Arabidopsis. Fluorescence was visualized by a confocal laser scanning microscope. Bar = 10μm. (B) Quantification of the subcellular localization pattern of AtVAMP727. The overlapping percentage of VAMP727:mCherry signal with GFP:AtVSR1 or GFP:SYP41, respectively, was determined from more than 50 protoplasts obtained from three independent experiments. Error bars represent SD, n = 3. **P≤0.01, by *t* test.

To this end, we examined the trafficking and subcellular distribution of SYP22 and VAMP727 by transient expression in *Arabidopsis* protoplasts (Figs 7 and 8).

For SYP22 assay, GFP gene was fused to the N-terminal end of *SYP22*, driven by the 35S promoter. The *SYP22*:*GFP* plasmid was transiently co-expressed in *Arabidopsis* leaf protoplasts with the PVC marker mRFP:AtVSR5, TGN marker RFP:SYP61 or Golgi marker Man1:RFP, respectively. SYP22:GFP fluorescence appeared on the punctate structures in the cytosol (Fig 7A). In Col-0, 79% of the SYP22:GFP fluorescent signals were co-localized with mRFP:AtVSR5, but only 10% with RFP:SYP61 and 11% with Man1:RFP, suggesting that the majority of SYP22 is localized to the PVC in Col-0 (Fig 7A and 7B). In *nhx5 nhx6*, however, the cellular distribution pattern of AtSYP22 was altered: 47% of SYP22:GFP overlapped with mRFP:AtVSR5, whereas 19% with SYP61and 31% with Man1:RFP, respectively (Fig 7A and 7B). These results suggest that a significant amount of SYP22 is trapped in the Golgi or TGN on the way to the PVC in the absence of AtNHX5 and AtNHX6. AtNHX5 and AtNHX6 are required for the localization of SYP22 in the PVC.

For VAMP727 assay, mCherry gene was fused to the C-terminal end of *VAMP727*, driven by the 35S promoter. The *VAMP727*:*mCherry* plasmid was transiently co-expressed in *Arabidopsis* leaf protoplasts with the PVC marker GFP:AtVSR1 and TGN marker GFP:SYP41, respectively. *VAMP727*:*mCherry* fluorescence appeared on the punctate structures in the cytosol (Fig 8A). In Col-0, 91% of VAMP727:mCherry overlapped with the PVC marker GFP:AtVSR1, only 9% with the TGN marker GFP:SYP41, suggesting that the majority of VAMP727 is localized to the PVC in Col-0 (Fig 8A and 8B). By contrast, in *nhx5 nhx6*, 61% of VAMP727:mCherry overlapped with the PVC marker GFP:AtVSR1, while 38% of VAMP727:mCherry overlapped with the TGN marker GFP:SYP41 (Fig 8A and 8B). These results suggest that the PVC localization of VAMP727:mCherry is considerately repressed in *nhx5 nhx6*. A significant amount of VAMP727 is trapped in the TGN on the way to the PVC in the absence of AtNHX5 and AtNHX6.

### AtNHX5 and AtNHX6 are co-localized with SYP22 and VAMP727

**Fig 9:**
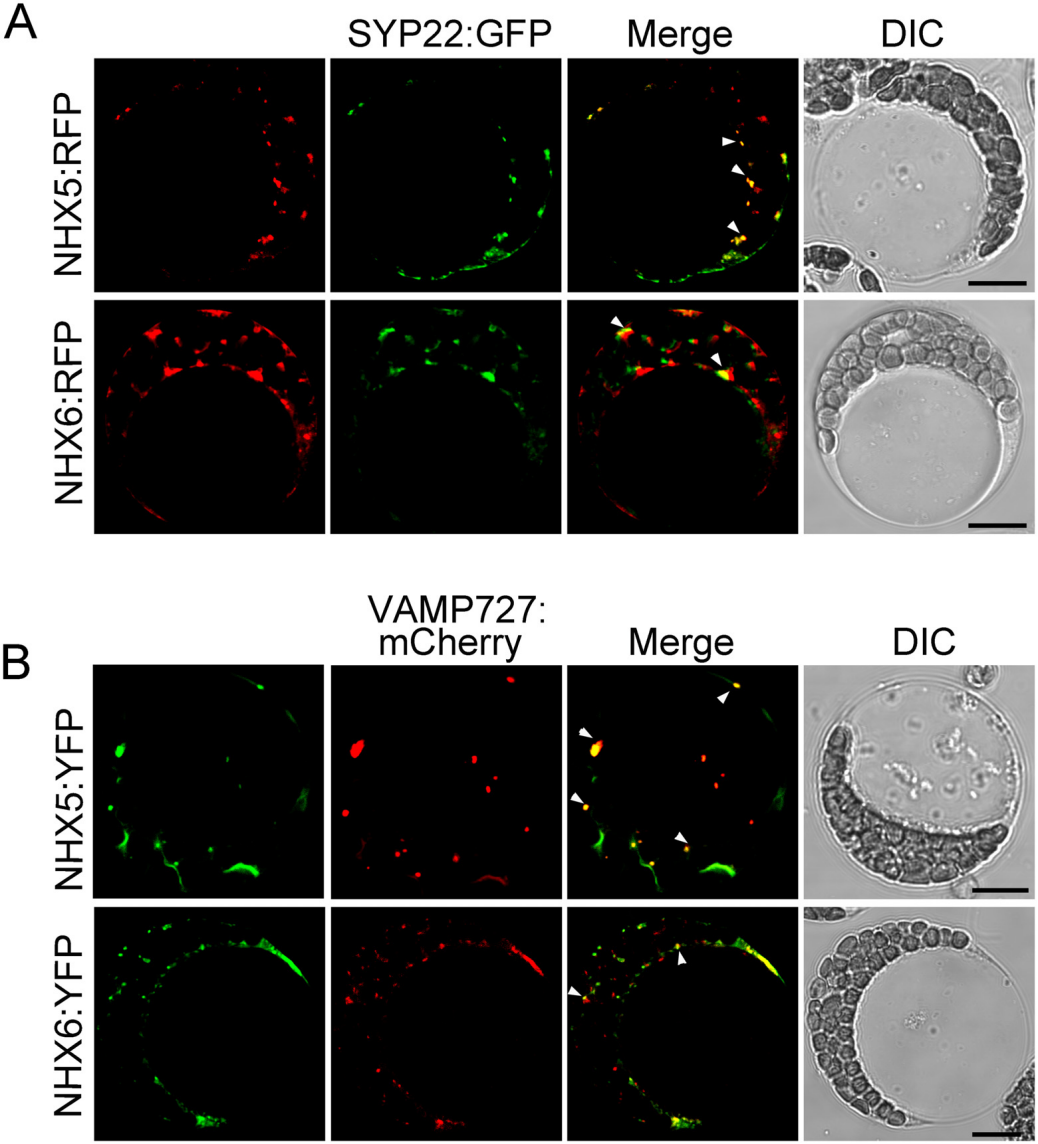
AtNHX5 and AtNHX6 are co-localized with SYP22 and VAMP727. (A) Subcellular localization of RFP:NHX5 or RFP:NHX6 with SYP22:GFP. The *RFP*:*AtNHX5* or *RFP*:*AtNHX6* plasmids were transiently co-expressed in *Arabidopsis* leaf protoplasts with SYP22:GFP, respectively. Fluorescence was visualized by a confocal laser scanning microscope. (B) Subcellular localization of YFP:NHX5 or YFP:NHX6 with VAMP727:mCherry. The *YFP*:*AtNHX5* or *YFP*:*AtNHX6* plasmids were transiently co-expressed in *Arabidopsis* leaf protoplasts with VAMP727:mCherry, respectively. Fluorescence was visualized by a confocal laser scanning microscope. Bar = 10 μm in (A) and (B).

We examined the colocalization of AtNHX5 and AtNHX6 with the SNAREs by transient expression in *Arabidopsis* protoplasts. *AtNHX5* and *AtNHX6* were tagged at the N-terminal ends with RFP and YFP, respectively, driven by the 35S promoter. The *RFP*:*AtNHX5* or *RFP*:*AtNHX6* plasmids were transiently co-expressed in *Arabidopsis* leaf protoplasts with SYP22:GFP, while the *YFP*:*AtNHX5* or *YFP*:*AtNHX6* plasmids were co-expressed with VAMP727:mCherry. As shown in Fig 9, RFP:AtNHX5 and RFP:AtNHX6 fluorescent signals partially co-localized with SYP22-GFP. In addition, YFP:AtNHX5 and YFP:AtNHX6 fluorescent signals were partially co-localized with VAMP727:mCherry. These results suggest that AtNHX5 and AtNHX6 are co-localized with SYP22 and VAMP727.

### AtNHX5 and AtNHX6 do not interact physically with the SNARE complex

**Fig 10:**
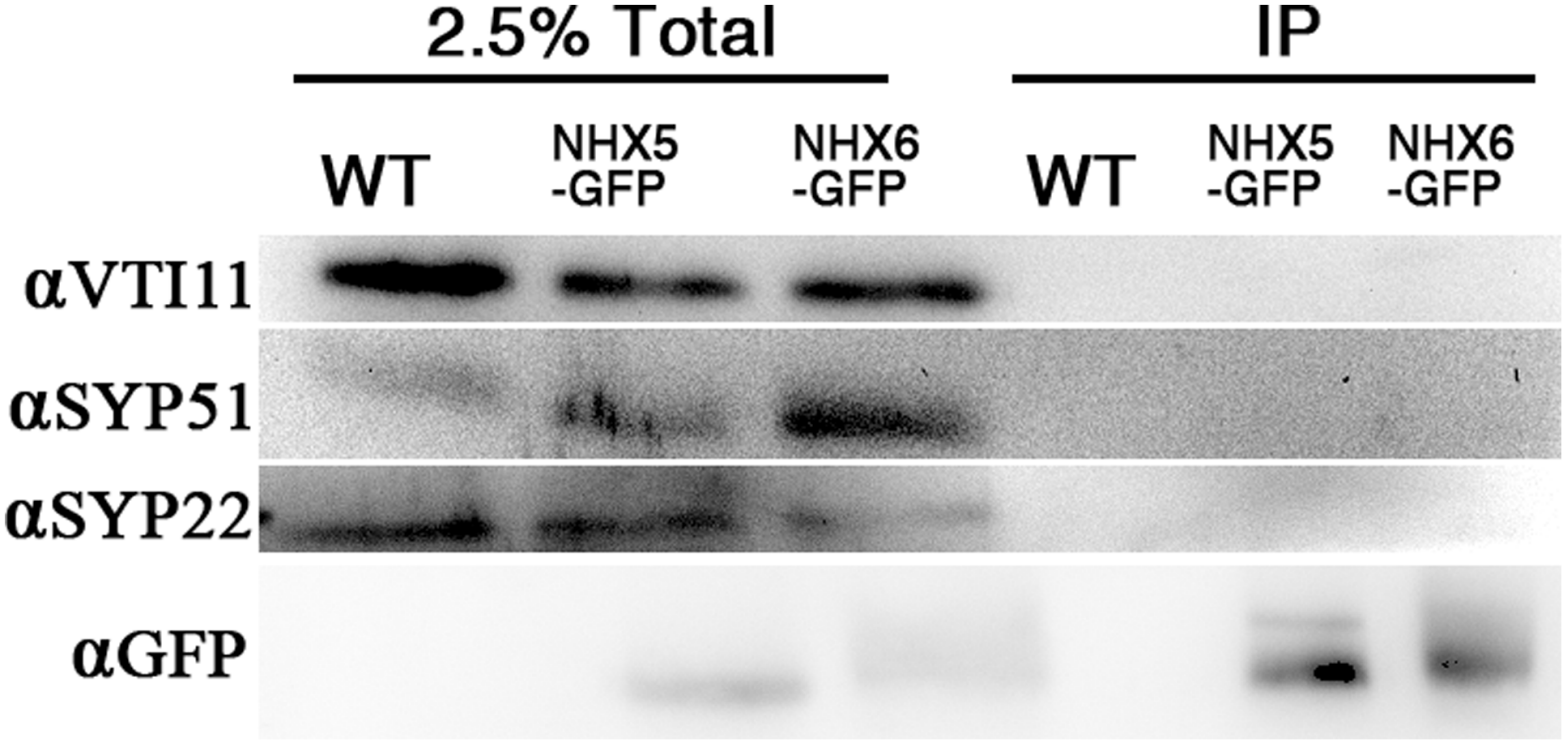
AtNHX5 and AtNHX6 do not interact physically with the SNARE complex. The transgenic *Arabidopsis* seedlings expressing GFP-tagged NHX5 or NHX6 were generated in our previous study (Wang et al., 2015). Anti-GFP monoclonal antibody was added to the plant lysate to precipitate NHX5-GFP or NHX6-GFP and their binding proteins. Coimmuoprecipitation of the SNARE proteins was analyzed with immunoblot with antibodies against SYP22, VTI11, or SYP51.

We next examined whether AtNHX5 or AtNHX6 interacted physically with the SNARE complex by coimmunoprecipitation (Fig 10).

We performed the coimmunoprecipitation using the transgenic *Arabidopsis* seedlings expressing GFP-tagged NHX5 or NHX6 that were generated in our previous study [49]. GFP was fused with the C-terminus of the *AtNHX5* or *AtNHX6* genes under the control of 35S promoters. The constructs were introduced into the *nhx5 nhx6* and wild-type plants. The chimeric genes coding for NHX5-GFP or NHX6-GFP functioned well since transformation of *nhx5 nhx6* with these constructs rescued the mutant phenotype [49]. The wild-type plants expressing GFP-tagged NHX5 or NHX6 were used to conduct the assay. Anti-GFP monoclonal antibody was added to the plant lysate to precipitate NHX5-GFP or NHX6-GFP and their binding proteins, followed by immunoblotting with antibodies against SYP22, VTI11, or SYP51. As shown in Fig 10, SYP22, VTI11 and SYP51 were not co-immunoprecipitated by the anti-GFP antibody, indicating that there was no physical interreaction between the SNARE complex and AtNHX5 or AtNHX6.

## Discussion

### AtNHX5 and AtNHX6 regulate the subcellular localization of the SNARE complex in *Arabidopsis*

In this report, we examined the role of AtNHX5 and AtNHX6 in the trafficking of the seed storage proteins to the PSVs. Using genetics, cellular and molecular techniques, we found that: (1) seed growth and production were altered in *nhx5 nhx6* double mutants (Fig 1); (2) transport of seed storage proteins into the PSVs was impaired in *nhx5 nhx6* (Fig 2, S1 and S2 Figs); (3) PSV biogenesis was changed in *nhx5 nhx6* (S3 Fig). These results indicate that AtNHX5 and AtNHX6 are involved in seed production, protein trafficking and PSV biogenesis, which are similar to what Reguera et al. (2015) had reported [39]. Together, both Reguera et al. (2015) and our results demonstrate that AtNHX5 and AtNHX6 are required for the transport of the seed storage proteins into the PSVs as well as the biogenesis of the PSVs in *Arabidopsis* [39].

It has been reported that the SNARE complex, which is composed of VAMP727, SYP22, VTI11 and SYP51, is critical for protein trafficking and PSV biogenesis in *Arabidopsis* [26]. This SNARE complex is localized to PVC to mediate the fusion between the PVC and the vacuole, through which proteins are delivered into the vacuole [26]. It is interesting to mention that AtNHX5 and AtNHX6 are localized to the Golgi, TGN and PVC, and these organelles are critical for protein trafficking to the vacuole in plants [39, 48, 55, 56]. There is great potential, therefore, for AtNHX5 and AtNHX6 to regulate the trafficking and subcellular distribution of this SNARE complex and hence its function in protein transport. We examined the role of AtNHX5 and AtNHX6 in regulating this SNARE complex. We found that: (1) seedling growth and seed development were altered in *nhx5 nhx6 syp22* (Fig 4); (2) PSV biogenesis was changed in *nhx5 nhx6 syp22* (Fig 5); (3) protein trafficking into the PSVs was impaired in *nhx5 nhx6 syp22* (Fig 6); (4) the localization of this SNARE complex in the PVC was repressed in the *nhx5 nhx6* (Figs 7 and 8). Our results indicate that AtNHX5 and AtNHX6 are required for the subcellular localization of this SNARE complex and hence its function in protein transport. Collectively, these results indicate that AtNHX5 and AtNHX6 may control the trafficking of the seed storage proteins to the PSVs through regulating the subcellular localization of the SNARE complex in *Arabidopsis*.

### The transport activity of AtNHX5 and AtNHX6 is required for the trafficking and subcellular localization of the SNARE complex

We further examined how AtNHX5 and AtNHX6 regulated the subcellular localization of the SNARE complex: via transport activity, physical interaction, or both. Studies from bacteria, yeast and mammals show that the acidic amino acid residues in transmembrane domains of Na^+^/H^+^ antiporters are critical for exchange activity [57–59]. Mutation of these conserved acidic residues in yeast ScNhx1p blocked protein trafficking in yeast. Our previous study has shown that AtNHX5 and AtNHX6 contain the conserved acidic amino acid residues in transmembrane domains that align with the yeast Na^+^/H^+^ antiporter ScNhx1p [49]. We have shown that three of these conserved acidic residues of AtNHX5 (D164, E188, and D193) and AtNHX6 (D165, E189, and D194) are essential for the ion transport activity of AtNHX5 and AtNHX6, and play an important role in growth and development in *Arabidopsis* [49]. In this study, we found that the precursor proteins of both 12S globulin and 2S albumin were detected for three of the point mutations in both *AtNHX5* and *AtNHX6* genes (D164N, E188Q and D193N of AtNHX5, and D165N, E189Q and D194N of AtNHX6) (Fig 3A and 3B), indicating that the transport activity of AtNHX5 and AtNHX6 is critical for protein transport into the PSVs. These results suggest that AtNHX5 and AtNHX6 may regulate the subcellular localization of the SNARE complex by their transport activity.

AtNHX5 and AtNHX6 are H^+^-coupled cotransporters whose biochemical activity is transferring the Na^+^ or K^+^ across a membrane in exchange for protons (H^+^). Thus, AtNHX5 and AtNHX6 play an important role in pH and ion homeostasis in *Arabidopsis* [39, 48, 49]. Our finding that the localization of the SNARE complex was regulated by the transport activity of AtNHX5 and AtNHX6, therefore, suggests that the localization of the SNARE complex was regulated by the pH and/or ion homeostasis maintained by AtNHX5 and AtNHX6. This is supported by the recent studies which show that AtNHX5 and AtNHX6 are vital for the endosomal pH homeostasis and this pH homeostasis is required for protein trafficking to the vacuole in *Arabidopsis* [31, 39].

To determine whether AtNHX5 or AtNHX6 physically interact with the SNARE complex, we performed coimmunoprecipitation assay. We found that SYP22, VTI11 and SYP51 were not co-immunoprecipitated by the anti-GFP antibody, suggesting that there is no physical interrelation between the SNARE complex and AtNHX5 or AtNHX6 (Fig 10).

### AtNHX5 and AtNHX6 have diversified roles in protein trafficking

In this report, we demonstrate that AtNHX5 and AtNHX6 control the trafficking of seed storage proteins by regulating the subcellular localization of the SNARE complex (Figs 7 and 8). On the other hand, Reguera et al. (2015) found that AtNHX5 and AtNHX6 controlled the trafficking of seed storage proteins by regulating the interaction between VSR and its cargoes [39]. Ashnest et al. (2015) found that AtNHX5 and AtNHX6 might regulate the recycling of VSRs by interacting with SNX1, a component of the Retromer [40]. Therefore, the results of Reguera et al. (2015), Ashnest et al. (2015) and ours revealed three distinct mechanisms for the role of AtNHX5 and AtNHX6 in protein trafficking: regulating the SNARE complex that mediates the fusion between the PVC and the vacuole, regulating the binding of VSR with its cargoes at the TGN, and regulating the recycling of VSRs from the TGN back to the ER. AtNHX5 and AtNHX6, thus, regulate three distinct steps of the protein trafficking pathway. In other words, AtNHX5 and AtNHX6 may have diversified roles in protein trafficking.

Why AtNHX5 and AtNHX6, then, have such diversified roles in protein trafficking? The short answer is that they have unique subcellular localizations in cells. We know that, in plant cells, proteins are transported to the vacuole through a vesicle-mediated trafficking pathway that includes the ER, Golgi, TGN, and MVB/PVC [50]. Thus, the Golgi, TGN and MVB/PVC are major protein sorting stations in vacuolar transport [55, 56]. Since AtNHX5 and AtNHX6 are localized to the Golgi, TGN and PVC [39], these two antiporters may regulate the protein trafficking activities carried out in these organelles, including the function of the SNARE complex and the receptor protein VSR. Therefore, AtNHX5 and AtNHX6 have such diversified roles in protein trafficking.

## Materials and Methods

### Plant materials and growth conditions

*Arabidopsis thaliana* ecotypes Columbia (Col-0), mutant and transgenic lines were used in this study. In the growth chamber, plants were grown on compost (Pindstrup Substrate, Latvia) and subirrigated with tap water. Greenhouse conditions were as follows: 16-h-light /8-h-dark cycles, light intensity 100 μmol s^-1^ m^-2^ photosynthetically active radiation, temperature 22°C, and relative humidity 50±10%.

For plate-grown plants, *Arabidopsis* thaliana seeds were surface sterilized with 20% (v/v) bleach. After cold treatment at 4°C for 3 days in the dark, the seeds were germinated on plates with Murashige and Skoog (MS) medium containing 1.0% agar, pH 5.8.

For silique phenotype observation, photos were taken at 22 DAF (DAF, days after flowering). The length of siliques on the main stem was measured, where the fifth to eighth siliques were measured.

To observe the developing embryo, the embryos were stripped from siliques at 14 DAF. For morphology observation of the mature seeds, photos were taken when the seeds were dehydrated for 30 days after harvest.

### Generation of the *nhx5 nhx6 syp22* triple mutant

T-DNA insertion lines were obtained from the SALK collection and GABI collection. T-DNA lines used in this work were Wisc-DsLox345-348M8 (*nhx5-1*), GABI_363E02 (*nhx5-2*), SALK_113129C (*nhx6-1*), SALK_100042 (*nhx6-2*), SALK_ 060946C (*syp22-1*) and 075924 (*syp22-3*). Insertion mutant information was obtained from the SIGnAL website (http://signal.salk.edu/cgi-bin/tdnaexpress) and confirmed experimentally. Positions of *nhx5-1*, *nhx6-1* and *nhx6-2* T-DNA insertion sites have been shown in Wang et al. (2015) [49]. The T-DNA insertion sites of *syp22-1*, *syp22-3* and *nhx5-2* are shown in S4A Fig online. The T-DNA insertion in line *syp22-1* occurred at nucleotide +1058 relative to the start codon, whereas mutant *syp22-3* carries the insertion at nucleotide +154 (S4A Fig). T-DNA insertion in line *nhx5-2* occurred at nucleotide +1371bp relative to the start codon (S4A Fig). Homozygous mutant lines were identified by PCR screening with allele-specific primers designed to amplify wild-type or mutated loci. The primers used for homozygous identification were as follows: *syp22-1* (SALK_060946C LP: 5’- TTTGAGCTCAAAGGGTGAATC-3’; SALK_060946C RP: 5’- TGCTGCTGAAAGAGAAACCAC-3’; BP LB1.3: 5’- ATTTTGCCGATTTCGGAAC-3’); *syp22-3* (SALK_075924 LP: 5’- TTAAACTAAGGGCACCGAACC-3’; SALK_075924 RP: 5’- AAAGTCCCTTGCAAGCTTAGC-3’; BP LB1.3: 5’- ATTTTGCCGATTTCGGAAC-3’); *nhx5-2* (GABI_363E02 LP: 5’- CAATTGCTCCGAAGACTTCAG-3’; GABI_363E02 RP: 5’- TTGGCACATCGATAGGACTTC -3’; GABI BP: 5’- ATAATAACGCTGCGGACATCTACAT -3’)

The *nhx5 nhx6* double mutant line, *nhx5-1 nhx6-1* was generated in our previous study [49]. The *nhx5-2 nhx6-2* was generated by crossing *nhx5-2* and *nhx6-2* (S4B Fig).

The triple mutant lines were generated by crossing *nhx5-1 nhx6-1* with *syp22-3*, or crossing *nhx5-2 nhx6-2* with *syp22-1*, using *nhx5-1 nhx6-1* and *nhx5-2 nhx6-2* as pollen donors. The homozygous triple mutant lines were identified by PCR screening with allele-specific primers designed to amplify wild-type or mutated loci.

### Transformation of SP-GFP-CT24 into *Arabidopsis*

The plasmid SP-GFP-CT24 was kindly provided by Dr. Shigeru Utsumi of Kyoto University, Japan. Transformation of *Arabidopsis* was performed by floral dipping using *Agrobacterium tumefaciens* (strain GV3101) [60]. The transformed seedlings (T1 plants) were selected on MS agar plates containing 200 μg ml^-1^ carbenicillin and 30 μg ml^-1^ kanamycin. The antibiotic positive seedlings were re-confirmed with PCR amplification of the GFP fragment using the following primers: GFP F: 5’- GTGAGCAAGGGCGAGGAGCTGTTC -3’, and GFP R: 5’- CAGCTCGTCCATGCCGAGAGTGATC -3’. The T2 seeds were collected and used for the experiments.

### Confocal laser scanning microscopy

The confocal laser scanning analysis was performed as described [10]. T2 seeds of the transformation plants were soaked for 20 minutes in PBS or distilled water. Then the cotyledons were peeled off from the seed coat. To observe the fluorescence, the cotyledons were placed on the glass slides, added a drop of glycerol, and covered with glass strips. Fluorescence was visualized by a confocal laser scanning microscope (FV1000, Olympus). The excitation wavelength was 488 nm for GFP and 594 nm for autofluorescence of PSVs, and emission was 500–530 nm for GFP and 590–630 nm for autofluorescence of PSVs. Images were processed with the Adobe Photoshop 3.0 (Adobe System). For quantitative analysis, 100 cells of the hypocotyls were measured in indicated genotypes seeds. Images were processed with Image J software to measure the size and numbers of the PSVs.

### Seed protein extraction

Seed proteins were extracted according to Nishizawa et al. (2003) with minor modifications [51]. Seeds were harvested and dehydrated for 30 days before protein extraction. Seeds were homogenized at 4°C in extraction buffer (150–200 μL extraction buffer/mg seeds). The extraction buffer contained 35 mM sodium phosphate buffer (pH 7.6), 0.4 M NaCl, 1 mM EDTA, 0.02% (w/v) NaN_3_, 0.1 mM pepstatin A, 10 mM 2-mercaptoethanol, and 0.1 mM (p-amidinophenyl) methanesulfonyl fluoride (p-APMSF). The homogenates were centrifuged at 13,000 × g for 15 min at 4°C to remove the cellular debris. The supernatants were collected as the crude protein extracts. The total protein of the crude extracts was determined with the Bradford protein assay kit (Sangon Biotech, China).

### SDS-PAGE and immunoblot analysis

Total protein (10 μg) of the supernatants was mixed with 2 μL 5× loading buffer composed of 250 mM Tris-HCl (pH = 6.8), 10% (W/V) SDS, 0.5% (W/V) Bromophenol Blue, 50% (V/V) glycerol and 5% (V/V) β-mercaptoethanol, then added protein extraction buffer to a total volume of 15 μL. The samples were boiled for 5 minutes and subjected to SDS-PAGE on 12% polyacrylamide gel. The separated proteins on gels were transferred electrophoretically to a polyvinylidene difluoride filter (0.45 μm) in a wet-electroblotting system, the membrane were blocked at room temperature for an hour with blocking solution containing 5% (w/v) skim milk dissolved in TBST (0.1 M Tris-HCl, pH 8.0, 0.15 M NaCl, 0.05% (w/w) Tween-20). Then the membrane was incubated with the primary antibodies against 12S albumin (diluted 10000-fold) or 2S globulin (diluted 10000-fold) in 1% (w/v) skim milk dissolved in TBST at 4°C overnight. After rinsed several times with TBST, the membrane was incubated in the horseradish peroxidase-conjugated goat antibody against rabbit IgG which was diluted 10000-fold in TBST plus 1% skim milk. Signals were detected with an ECL kit (Thermo Scientific).

The anti-12S globulin and anti-2S albumin antibodies were kindly provided by Dr. Shigeru Utsumi of Kyoto University, Japan. The second antibody (the horseradish peroxidase-conjugated goat antibody against rabbit IgG) was purchased from ZSGB-BIO, China.

### Generation of the point mutants of the conserved acidic residues in AtNHX5 and AtNHX6

The point mutants of the conserved acidic residues of AtNHX5 and AtNHX6 were generated from our previous study [49]. Briefly, pDONR-NHX5 and pDONR-NHX6 were used as templates to generate the point mutants. The site-directed mutagenesis was performed by Quikchange mutagenesis. For *AtNHX5*, the mutations were GAC to AAT (pDONR-NHX5-D164N), GAA to CAA (pDONR-NHX5-E188Q), GAT to AAT (pDONR-NHX5-D193N), and GAA to CAA (pDONR-NHX5-E320Q). The mutations of *AtNHX6* were GAT to AAT (pDONR-NHX6-D165N), GAA to CAA (pDONR-NHX6-E189Q), GAT to AAT (pDONR-NHX6-D194N), and GAG to CAA (pDONR-NHX6-E320Q). These point mutants of *AtNHX5* or *AtNHX6* were recombined into pUBC-GFP using Gateway technology. The C-termini of these genes was fused with GFP, driven by Ubiquitin-10 (Ub10) promoter. These plasmids were transformed into the GV3101 *A*. *tumefaciens* strain, and the resulting bacterial clones were used to transform the *A*. *thaliana* (ecotype Columbia) by the floral dip procedure [60]. Transgenic plants were screened *in vitro* on MS medium supplemented with 0.0015% Basta. The homozygous lines of T3 progeny were selected for experiments.

### Transient expression in *Arabidopsis* protoplasts and confocal microscopy

The PVC makers mRFP:AtVSR5 and GFP:AtVSR1, the TGN marker RFP:SYP61, and the Golgi marker Man1:RFP were generously provided by Dr. Liwen Jiang of The Chinese University of Hong Kong, China. The PVC/vacuoloar membrane marker GFP:SYP22 and the TGN marker GFP:SYP41 were kindly provided by Dr. Masa H. Sato of Kyoto University, Japan.

VAMP727 gene was constructed into the plasmid pSAT4A-mCherry-N1. mCherry gene was fused in frame to *VAMP727* at its C-terminus. The VAMP727 gene was amplified using the following primers: VAMP727 (5’-CGCGGATCCATGAGTCAAAAGGGT-3’ and 5’- TGCTCTAGATGATGAGCATTTGAAACCTC-3’). Then the amplified production was cloned into the BamH1 and Xba1 sites of the pSAT4A-mCherry-N1 vector to yield the final plasmid VAMP727: mCherry. The positive gene fragment was verified by sequencing.

RFP gene and YFP gene were fused in frame to the N-termini of *AtNHX5* and *AtNHX6*. The *AtNHX5* and *AtNHX6* were amplified using the following primers: AtNHX5 (5’-TCGCGGATCCATGGAGGAAGTGATGATT-3’ and 5’- CCCGGAATTCCTACTCCCCATCTCCATC-3’), AtNHX6 (5’- ATCGCGGATCCATGTCGTCGGAGCT-3’ and 5’- TCCGGAATTCTTAGCCGCGGTTATTTAGAT-3’). The amplified fragments were cloned into the EcoRI and BamHI sites of the pSAT6-C1-red fluorescent protein (RFP) and pSAT6-C1-yellow fluorescent protein (YFP) vector to yield the final plasmids RFP:AtNHX5, RFP:AtNHX6, YFP:AtNHX5 and YFP:AtNHX6.

The transient expression in protoplasts was performed as described [61, 62]. *Arabidopsis* seedlings of 4 weeks old were used for protoplast isolation. The protoplasts were derived from the leaf mesophyll cells of *Arabidopsis*. For SYP22 analysis, the plasmid GFP:SYP22 was transiently co-expressed in leaf protoplasts with the PVC marker mRFP:AtVSR5, TGN marker RFP:SYP61 and Golgi marker Man1:RFP. For VAMP727, VAMP727:mCherry was transiently co-expressed in leaf protoplasts with the PVC marker GFP:AtVSR1 and TGN marker GFP:SYP41. For co-localization assay between AtNHX5 or AtNHX6 and SYP22, the *RFP*:*AtNHX5* or *RFP*:*AtNHX6* plasmids were transiently co-expressed in *Arabidopsis* leaf protoplasts with SYP22:GFP. For co-localization assay between AtNHX5 or AtNHX6 and VAMP727, the *YFP*:*AtNHX5* or *YFP*:*AtNHX6* plasmids were transiently co-expressed in *Arabidopsis* leaf protoplasts with VAMP727:mCherry.

For confocal laser scanning microscopy, fixed cell were examined using object UPLAPO40×O13 (Olympus FV1000). The excitation/emission wavelengths were 488 /500-530 nm for GFP, 515/ 515–550 nm for YFP, and 559/590-630 for RFP, respectively. The colocalization was examined by directly monitoring the red, green or yellow fluorescence signal. To compare the images between Col-0 and *nhx5 nhx6*, the same conditions for image-taking and data analysis, including laser intensity, gray level, line average, and image processing, were used for Col-0 and *nhx5 nhx6*.

Quantitation of the colocalization of different fluorescence signals was performed as described [7,12]. The images of single frames from projection of the Z-stack were used for quantitation analysis. The images were disposed by Adobe Photoshop before quantitating the colocalization area with colocalization plugin in Image J (http://imagej.nih.gov/ij/plugins/).

### Crosslinking and immunoprecipitation

1.5 g of 12-day-old wild-type seedlings and transgenic seedlings expressing GFP-tagged NHX5 or NHX6 were incubated in crosslinking buffer (PBS pH 7.4 and 1 mM dithiobis (succinimidyl propionate))for 1 hr at room temperature (RT), and then 1M Tris-HCl (pH 7.5) was added (final concentration, 20 mM) for blocking and incubated for 30 min at RT. Samples were then grinded in 1 ml of grinding buffer (50 mM HEPES-KOH pH7.5, 5 mM MgCl_2_, 2 mM EGTA, 250 mM sorbitol, and protease inhibitor cocktail Complete, EDTA-free (Roche)), and centrifuged at 1,000 × g for 10 min to remove debris. Supernatants were centrifuged at 20,000 ×g for 10 min to collect membrane fraction, and pellets were applied to immunoprecipitation. Immunoprecipitation from detergent extracts was conducted using the micro-MACS GFP-tagged protein isolation kit (Miltenyi Biotec) according to the manufacturer's instructions.

### Electron microscopy

Sections were carried out as described with modifications [26, 50]. The *Arabidopsis thaliana* dry seeds were soaked in 50 mM PBS (pH 7.2) for 30 min, then, dozens of whole cotyledons were striped from the testa and fixed with buffer containing PBS (50 mM, pH 7.2) and 0.5% (w/v) glutaraldehyde for less than two hours, followed by dehydration in an ethanol series, and embedded in epoxy resin (Quetol-812; China).

For immunolabeling, sections were mounted on formvar-coated nickel grids etched with 3% (v/v) aqueous hydrogen peroxide for 10 min. After rinsing with distilled water several times, the ultrathin sections blocked for 30 min with blocking solution containing 5% (w/v) BSA and 0.1% Tween-20 in PBS (pH = 7.4). Then sections were incubated with antiserum against 12S globulin (1:50) diluted with diluting solution composed of PBS containing 0.25% Tween-20 and 1% (w/v) BSA at 4°C for overnight. After washing in diluting solution for three times, sections were incubated for 1hour at room temperature with antirabbit IgG secondary antibody conjugated to 15 nm colloidal gold (Sangon Biotech, China) diluted 1:20 in diluting solution. The sections were rinsed with PBS or distilled water and aired without stain. The sections were examined with a transmission electron microscope. Control experiment was performed by omitting the primary antibody.

### Accession numbers

The *Arabidopsis* Genome Initiative locus identifiers for the genes mentioned in this article are, NHX5 (At1g54370), NHX6 (At1g79610), VAMP727 (At3g54300), SYP22 (At5g46860), VSR1 (AT3G52850), VSR5 (AT2G34940), SYP41 (AT5G26980), SYP61 (AT1G28490) and Man1 (AT1G02310).

## Supporting Information

S1 Fig Precursors of the storage proteins are accumulated in the seeds of *nhx5 nhx6*. (A) SDS-PAGE stained with the Coomassie blue. Total proteins were extracted from the mature seeds. 10 μg Proteins were loaded in each lane. (B) and (C) Immunoblot analysis of 12S globulin (B) and 2S albumin (C).
(TIF)

S2 Fig 12S albumins were missorted out of the cells in embryos of *nhx5 nhx6*. (A) and (B) Immune EM assay. Embryos of Col-0 (A) and *nhx5 nhx6* (B) were labeled with 12S albumin antibody. Bars = 250 nm. (C) and (D) Enlarged photos showing the extracellular spaces in embryos. The 12S antibody labeled the extracellular space in the double mutant (arrowheads). CW, cell wall; L, lipid body. Bars = 1μm.
(TIF)

S3 Fig AtNHX5 and AtNHX6 play an important role in the biogenesis of the PSVs in Aabidopsis. (A) Morphology of the PSVs. Autofluorescence of the PSVs was visualized with a confocal laser scanning microscope. Bars = 10 μm. (B) Histograms representing a distribution of size and number of vacuoles within a single cell. Area of each vacuole was measured in cells of Col-0 and mutant embryos.
(TIF)

S4 Fig Molecular genetics analyses and growth phenotype of the *nhx5 nhx6 syp22* triple mutant. (A) T-DNA insertion sites in mutant alleles *nhx5-2*, *syp22-1* and *syp22-3*. Black boxes and lines represent the exons and introns, respectively, in the coding region. (B) The growth phenotype of *nhx5-2 nhx6-2 syp22-1* triple mutant. Seedlings were grown on soil for 30 d. (C) RT-PCR analysis of the mRNA expression level in the *nhx5-2 nhx6-2* double mutant line. ACT2 was used as a control. (D) RT-PCR analysis of the mRNA expression level in the triple mutant lines. ACT2 was used as a control. In the *nhx5-2 nhx6-2 syp22-1* triple mutant line, a cDNA fragment of SYP22, which was short than the WT, was detected by RT-PCR. This is consistent with the report of Ohtomo et al. (2005), where they showed that a 63 bp deletion was generated in *syp22* mutant. Both Ohtomo et al. (2005) and us used the same T-DNA insertion line SALK_ 060946C, which has a T-DNA insertion in the fifth exon of *SYP22*.
(TIF)
