# Supplementary figures and images for: AtNHX5 and AtNHX6 Are Required for the Subcellular Localization of the SNARE Complex That Mediates the Trafficking of Seed Storage Proteins in Arabidopsis
Source: PLoS One. 2016 Mar 17;11(3):e0151658. doi: 10.1371/journal.pone.0151658 (PMC4795774; doi:10.1371/journal.pone.0151658)

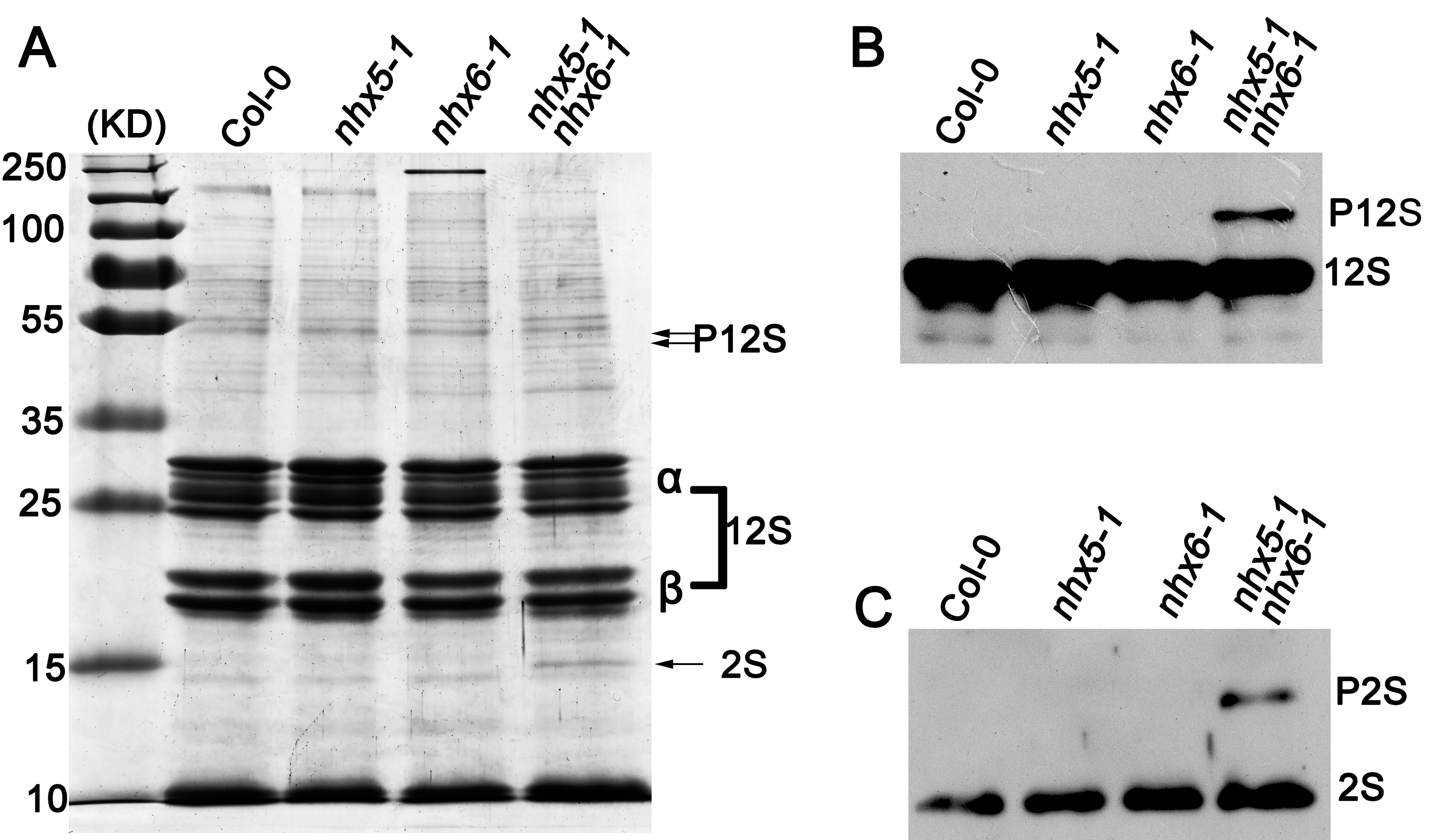

Supplement: S1 Fig — (A) SDS-PAGE stained with the Coomassie blue. Total proteins were extracted from the mature seeds. 10 μg Proteins were loaded in each lane. (B) and (C) Immunoblot analysis of 12S globulin (B) and 2S albumin (C). (TIF) [file pone.0151658.s001.tif]

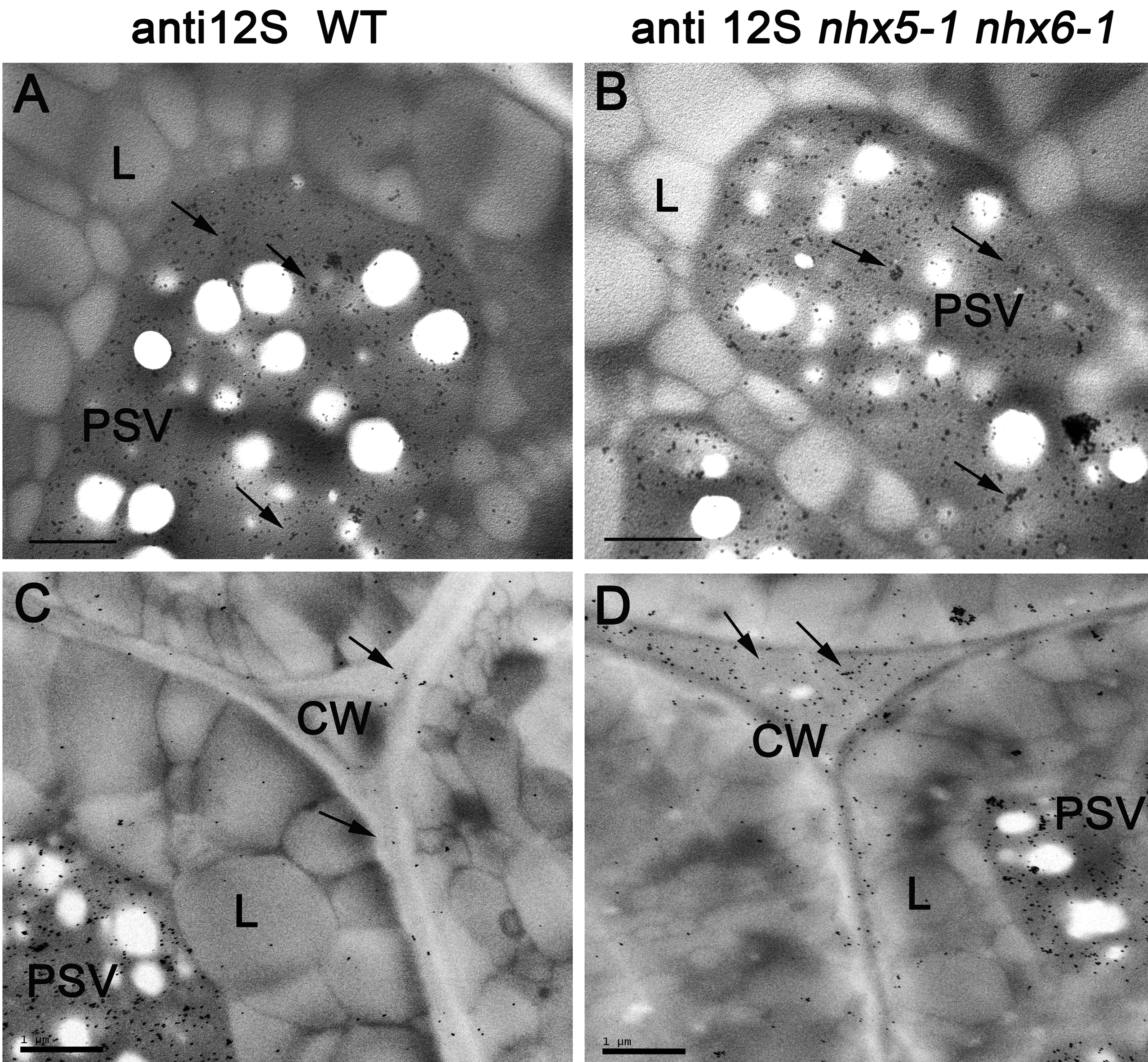

Supplement: S2 Fig — (A) and (B) Immune EM assay. Embryos of Col-0 (A) and nhx5 nhx6 (B) were labeled with 12S albumin antibody. Bars = 250 nm. (C) and (D) Enlarged photos showing the extracellular spaces in embryos. The 12S antibody labeled the extracellular space in the double mutant (arrowheads). CW, cell wall; L, lipid body. Bars = 1μm. (TIF) [file pone.0151658.s002.tif]

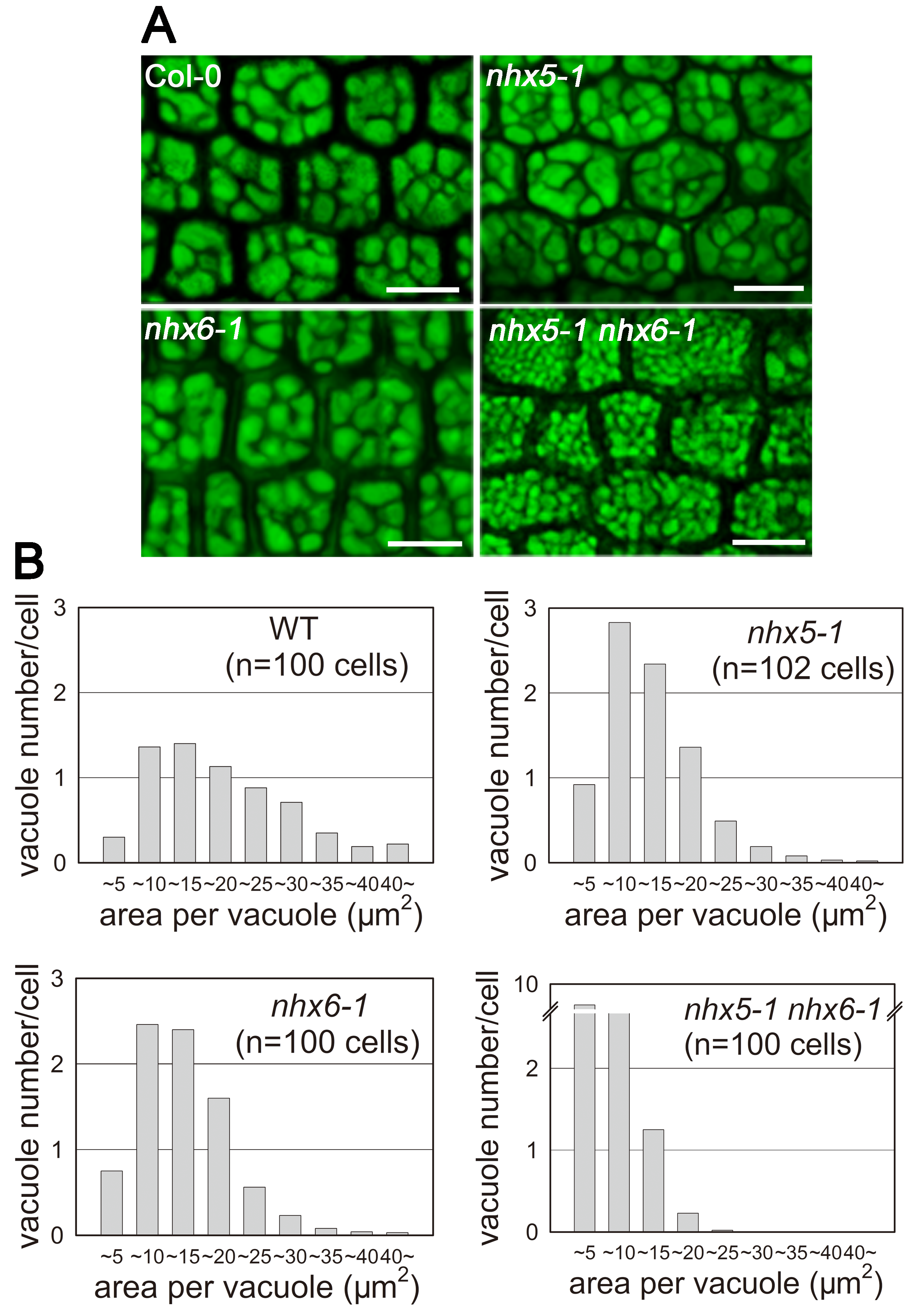

Supplement: S3 Fig — (A) Morphology of the PSVs. Autofluorescence of the PSVs was visualized with a confocal laser scanning microscope. Bars = 10 μm. (B) Histograms representing a distribution of size and number of vacuoles within a single cell. Area of each vacuole was measured in cells of Col-0 and mutant embryos. (TIF) [file pone.0151658.s003.tif]

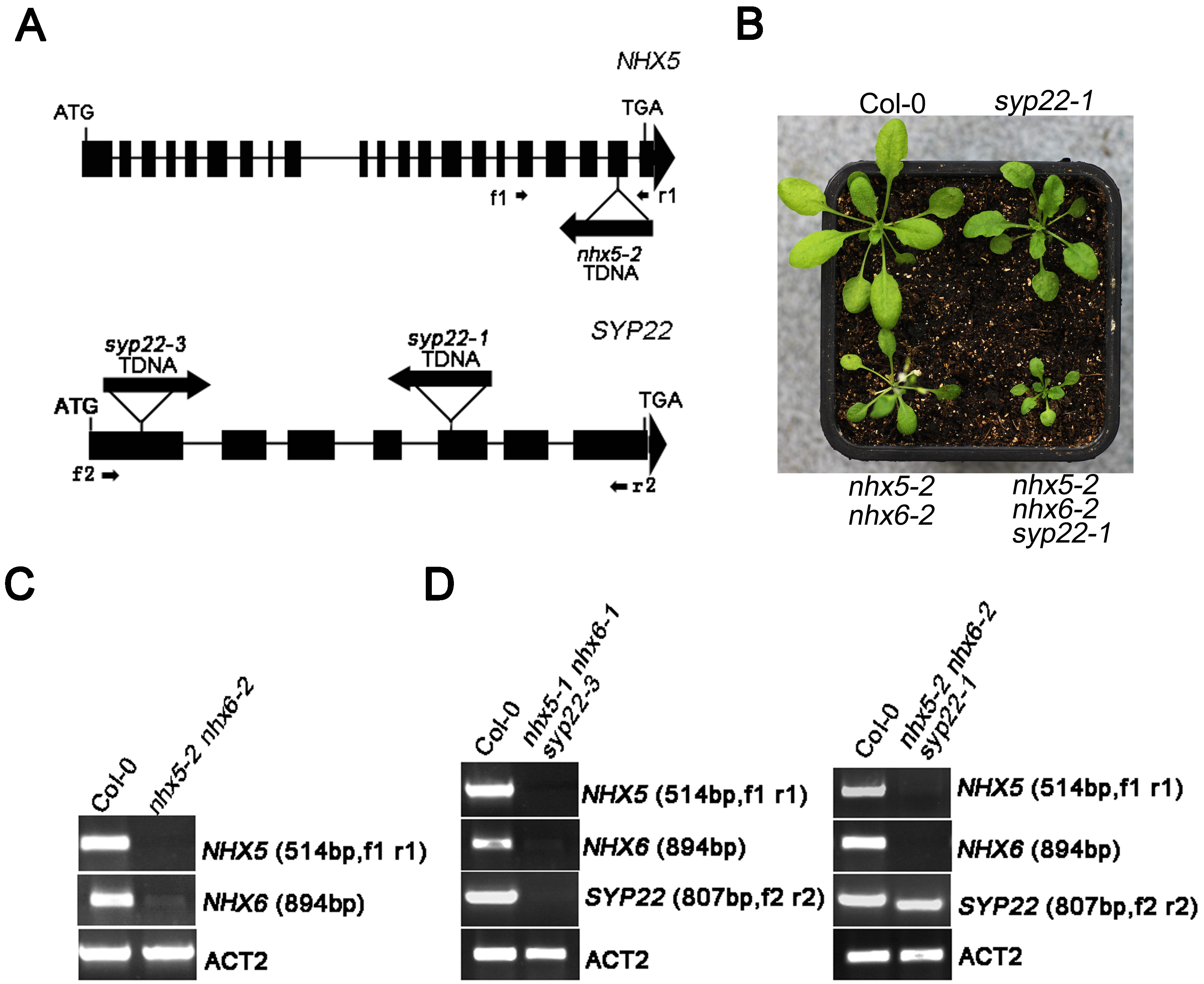

Supplement: S4 Fig — (A) T-DNA insertion sites in mutant alleles nhx5-2, syp22-1 and syp22-3. Black boxes and lines represent the exons and introns, respectively, in the coding region. (B) The growth phenotype of nhx5-2 nhx6-2 syp22-1 triple mutant. Seedlings were grown on soil for 30 d. (C) RT-PCR analysis of the mRNA expression level in the nhx5-2 nhx6-2 double mutant line. ACT2 was used as a control. (D) RT-PCR analysis of the mRNA expression level in the triple mutant lines. ACT2 was used as a control. In the nhx5-2 nhx6-2 syp22-1 triple mutant line, a cDNA fragment of SYP22, which was short than the WT, was detected by RT-PCR. This is consistent with the report of Ohtomo et al. (2005), where they showed that a 63 bp deletion was generated in syp22 mutant. Both Ohtomo et al. (2005) and us used the same T-DNA insertion line SALK_ 060946C, which has a T-DNA insertion in the fifth exon of SYP22. (TIF) [file pone.0151658.s004.tif]
